# A long and abundant non-coding RNA in *Lactobacillus salivarius*

**DOI:** 10.1099/mgen.0.000126

**Published:** 2017-07-17

**Authors:** Fabien J. Cousin, Denise B. Lynch, Victoria Chuat, Maxence J. B. Bourin, Pat G. Casey, Marion Dalmasso, Hugh M. B. Harris, Angela McCann, Paul W. O’Toole

**Affiliations:** ^1^​ School of Microbiology, University College Cork, Cork, Ireland; ^2^​ APC Microbiome Institute, University College Cork, Cork, Ireland; ^†^​ Present address: Normandie Univ, UNICAEN, EA4651 ABTE, F-14032 Caen, France.

**Keywords:** *Lactobacillus*, *L. salivarius*, non-coding RNA, RNA-seq, megaplasmid

## Abstract

*Lactobacillus salivarius*, found in the intestinal microbiota of humans and animals, is studied as an example of the sub-dominant intestinal commensals that may impart benefits upon their host. Strains typically harbour at least one megaplasmid that encodes functions contributing to contingency metabolism and environmental adaptation. RNA sequencing (RNA-seq)transcriptomic analysis of *L. salivarius* strain UCC118 identified the presence of a novel unusually abundant long non-coding RNA (lncRNA) encoded by the megaplasmid, and which represented more than 75 % of the total RNA-seq reads after depletion of rRNA species. The expression level of this 520 nt lncRNA in *L. salivarius* UCC118 exceeded that of the 16S rRNA, it accumulated during growth, was very stable over time and was also expressed during intestinal transit in a mouse. This lncRNA sequence is specific to the *L. salivarius* species; however, among 45 *L* *. salivarius* genomes analysed, not all (only 34) harboured the sequence for the lncRNA. This lncRNA was produced in 27 tested *L. salivarius* strains, but at strain-specific expression levels. High-level lncRNA expression correlated with high megaplasmid copy number. Transcriptome analysis of a deletion mutant lacking this lncRNA identified altered expression levels of genes in a number of pathways, but a definitive function of this new lncRNA was not identified. This lncRNA presents distinctive and unique properties, and suggests potential basic and applied scientific developments of this phenomenon.

## Abbreviations

asRNA, anti-sense RNA; HTH, helix-turn-helix; lasRNA, long anti-sense RNA; lncRNA, long non-coding RNA; ncRNA, non-coding RNA; OLE RNA, ornate, large, extremophilic RNA; qPCR, quantitative PCR; RNA-seq, RNA sequencing; RT-qPCR, reversetranscription-quantitative PCR; tmRNA, transfer-messenger RNA.

## Data Summary

1. Raw RNA-seq reads are available at the Sequence Read Archive (SRA) under BioProject; accession number: PRJNA355319 (url – https://www.ncbi.nlm.nih.gov/bioproject/PRJNA355319).

2. The GenBank accession number for the lncRNA sequence of *L. salivarius* UCC118 is MF114321.

3. The GenBank accession number for the lncRNA sequence of *L. salivarius* UCC119 is MF114322.

4. The GenBank accession number for the lncRNA sequence of *L. salivarius* AH4231 is MF114323.

5. The GenBank accession number for the lncRNA sequence of *L. salivarius* AH4331 is MF114324.

6. The GenBank accession number for the lncRNA sequence of *L. salivarius* AH43310 is MF114325.

7. The GenBank accession number for the lncRNA sequence of *L. salivarius* AH43324 is MF114326.

8. The GenBank accession number for the lncRNA sequence of *L. salivarius* AH43348 is MF114327.

9. The GenBank accession number for the lncRNA sequence of *L. salivarius* CCUG45735 is MF114328.

10. The GenBank accession number for the lncRNA sequence of *L. salivarius* CCUG47825 is MF114329.

11. The GenBank accession number for the lncRNA sequence of *L. salivarius* CCUG47826 is MF114330.

12. The GenBank accession number for the lncRNA sequence of *L. salivarius* L21 is MF114331.

13. The GenBank accession number for the lncRNA sequence of *L. salivarius* NCIMB8818 is MF114332.

14. The GenBank accession number for the lncRNA sequence of *L. salivarius* JCM1046 is MF114333.

15. The GenBank accession number for the lncRNA sequence of *L. salivarius* NCIMB8817 is MF114334.

16. The GenBank accession number for the lncRNA sequence of *L. salivarius* DSM20492 is MF114335.

17. The GenBank accession number for the lncRNA sequence of *L. salivarius* CCUG47171 is MF114336.

18. The GenBank accession number for the lncRNA sequence of *L. salivarius* CCUG44481 is MF114337.

19. The GenBank accession number for the lncRNA sequence of *L. salivarius* 01M14315 is MF114338.

20. The GenBank accession number for the lncRNA sequence of *L. salivarius* CCUG43299 is MF114339.

21. The GenBank accession number for the lncRNA sequence of *L. salivarius* JCM1040 is MF114340.

22. The GenBank accession number for the lncRNA sequence of *L. salivarius* DSM20555^T^ is MF114341.

23. The GenBank accession number for the lncRNA sequence of *L. salivarius* Gul1 is MF114342.

24. The GenBank accession number for the lncRNA sequence of *L. salivarius* Gul2 is MF114343.

25. The GenBank accession number for the lncRNA sequence of *L. salivarius* JCM1047 is MF114344.

26. The GenBank accession number for the lncRNA sequence of *L. salivarius* CCUG38008 is MF114345.

27. The GenBank accession number for the lncRNA sequence of *L. salivarius* LMG14476 is MF114346.

28. The GenBank accession number for the lncRNA sequence of *L. salivarius* LMG14477 is MF114347.

29. Microarray data were submitted to the National Center for Biotechnology Information into the Gene Expression Omnibus (GEO) database under pending accession number GSE92837 (url – https://www.ncbi.nlm.nih.gov/geo/query/acc.cgi?token=kzynyucqxvaldgj&acc=GSE92837).

## Impact Statement

The role of non-coding RNAs (ncRNAs) in regulating cellular processes in prokaryotes is relatively under-investigated. The present study identifies a new long non-coding RNA (lncRNA) in the gut commensal species *Lactobacillus salivarius*. This lncRNA is expressed at very high abundance, occasionally exceeding that of the 16S rRNA gene. The lncRNA is specific to the *L. salivarius* species, but not all strains harbour it in their genomes, and its expression level is strain-specific. High-level lncRNA expression correlates with high megaplasmid copy number. This lncRNA presents distinctive and unique properties, and suggests potential basic and applied scientific developments. These discoveries expand our appreciation of the ncRNA world in bacteria.

## Introduction

*Lactobacillus salivarius* is commonly found in the gastrointestinal tract of human and animals, and has been studied particularly in the context of beneficial effects on the host [1, 2]. Analysis of traits of interest, such as resistance to bile, production of bacteriocin and exopolysaccharide, has been facilitated by the establishment of genomic and genetic tools in strain UCC118 [3, 4].

Forty-five genome sequences of *L. salivarius* have recently been annotated in our laboratory [5]. A typical *L. salivarius* genome presents one chromosome, one (and rarely two) megaplasmid(s) and up to three small plasmids depending on the strain [6]. The megaplasmid seems indispensable for the viability of *L. salivarius*, and presents strain-specific characteristics, including size and coding repertoire [4, 6]. The megaplasmid pMP118 in strain UCC118 harbours genes playing roles in environmental adaptation. The presence of this megaplasmid may explain the relatively small size (1.83 Mb) of the *L. salivarius* chromosome, because some structural genes are harboured by the megaplasmid.

Data from the increasing number of bacterial genomes and NGS transcriptomic studies have indicated that non-coding RNAs (ncRNAs) play an important role in the regulation of function of all bacterial replicons, like chromosomes, plasmids or transposons [7–9]. Many studies of Gram-positive pathogens identified the regulation of virulence by ncRNA, such as that which occurs in *Staphylococcus aureus* [10–12] and *Listeria monocytogenes* [13–15]. ncRNAs include anti-sense RNAs (asRNAs), intergenic small RNAs, riboswitches and long non-coding RNAs (lncRNAs) [16–20]. lncRNAs are characterized by a particular size presumed necessary for their function (>200 nt), and can regulate one set of genes; however, their functions remain largely unknown.

Several new long regulatory RNAs have been described in the past decade, including the long regulatory OLE RNA (ornate, large, extremophilic RNA) of 610 nt [21], the lncRNA GOLLD RNA (giant, ornate, lake- and lactobacillales-derived RNA) of ∼800 nt and the lncRNA HEARO (HNH endonuclease-associated RNA and ORF) [22]. These long anti-sense RNAs (lasRNAs) appear to be more complex than other ncRNAs and their activity is not limited to regulation. Although the exact function of these lncRNAs is not currently known, it was demonstrated for the OLE RNA that the protein encoded by the gene downstream of this lncRNA can bind the OLE RNA to form a complex. This nucleoprotein plays an important role in stress response by binding to the cellular membrane and enhancing membrane resistance towards short chain alcohols [23, 24].

Recently, many lasRNAs were also described in *L. monocytogenes* [18, 25] suggesting that the biological significance of lasRNAs has been underestimated up to now. New lncRNAs were also found in *Lactobacillus plantarum*, with lengths ranging from 800 nt up to 1000 nt [26]. Although their precise function is still unknown, these *L. plantarum* supermotifs (LPSMs) are conserved, with many copies present across strains in this species. However, LPSMs seem very specific to *L. plantarum*, as they were not found in the genomes of other *Lactobacillus* species [26]. Transfer-messenger RNA (tmRNA), previously named 10S RNA, is also known as a lncRNA (260 to 430 nt). tmRNAs take part in protein translation by ensuring protein quality. When the ribosome is stalled, tmRNAs add a protease-recognition tag to the incomplete protein to allow its degradation [27–30]. tmRNA is very conserved and is present in all bacteria, which makes it a good reference for the investigation of lncRNA.

In this study, we describe an unusually abundant and stable lncRNA that we identified by RNA sequencing (RNA-seq)analysis of *L. salivarius* UCC118. This lncRNA is a unique feature of *L. salivarius* and its coding sequence was present in 34 of 45 *L* *. salivarius* genomes available. The lncRNA was expressed in all 27 tested *L. salivarius* strains harbouring the sequence, with strain-specific expression levels that varied by almost 4 logs between strains. Four *L. salivarius* strains expressed this lncRNA at a very high expression level and only these strains presented a megaplasmid : chromosome ratio greater than 1.0. The analysis of knock-out mutants in which the lncRNA sequence was removed did not clearly identify the role of this new lncRNA.

## Methods

### Bacterial strains, growth conditions and growth measurement

We assembled a panel of 45 *L* *. salivarius* genomic sequences, 33 of which were available in our laboratory strain collection (Table 1). All strains were inoculated at 1 % from an overnight culture in pre-warmed (37 °C)MRS(De Man, Rogosa and Sharpe) broth medium (Oxoid) and incubated (37 °C, 5 % CO_2_). Optical density was measured at 600 nm (Spectramax; Molecular Devices). Growth rate was calculated by the linear regression of ln(OD_600_) for each point during the exponential phase. Each experiment was performed in duplicate.

**Table 1.** Strains of *L. salivarius* used in this study Strain L21 was provided by Professor Gerald Tannock, University of Otago, Otago, New Zealand. The 11 strains highlighted in grey share 100 % identity at the nucleotide level for the lncRNA. A question mark (?) indicates that the size of the megaplasmid is currently unknown. The *L. salivarius* strains were divided into 4 groups: +++++, for the strains with very high expression of the lncRNA; +++, for the strains with sequence 100 % identical to the *L. salivarius* UCC118 lncRNA; +, for the strains harbouring a lncRNA sequence with SNPs/gaps; −, for the strains without a lncRNA sequence in their genome. CCUG, Culture Collection University Göteborg; DSM, Deutsche Sammlung von Mikroorganismen und Zellkulturen; JCM, Japan Collection of Microorganisms; LMG, Laboratorium voor Microbiologie, Universiteit Gent; NCIMB, National Collections of Industrial Food and Marine Bacteria.

**Strain name**	**Source**	**Megaplasmid size (kb)**	**lncRNA alignment length**	**lncRNA** **% identity**	**lncRNA group**
UCC118	Human ileal–caecal region	242	520	100	+++++
UCC119	Chicken caecum	195	520	100	+++++
AH4231	Human ileal–caecal region	210	520	100	+++
AH4331	Human ileal–caecal region	210	520	100	+++
AH43310	Human ileal–caecal region	240	520	100	+++++
AH43324	Human ileal–caecal region	240	520	100	+++++
AH43348	Human ileal–caecal region	195	520	100	+++
CCUG45735	Human blood	220	520	100	+++
CCUG47825	Human blood	220	520	100	+++
CCUG47826	Human blood	220	520	100	+++
L21	Human faeces	190	520	100	+++
NCIMB8818	St Ivel cheese	195	520	99.81	+
JCM1046	Swine intestine	230	521	98.46	+
NCIMB8817	Turkey faeces	145	521	98.08	+
DSM20492	Human saliva	240	520	97.88	+
CCUG47171	Human tooth plaque	240	521	97.12	+
CCUG44481	Bird	240	520	97.5	+
01M14315	Human gallbladder pus	200	521	97.5	+
CCUG43299	Human blood	218	521	97.5	+
JCM1040	Human intestine	195	521	97.5	+
DSM20555**^T^**	Human saliva	380	528	96.02	+
Gul1	Root canal	?	528	96.02	+
Gul2	Root canal	?	528	96.02	+
JCM1047	Swine intestine	240	521	97.31	+
CCUG38008	Human gall	215	521	95.97	+
LMG14476	Cat with myocarditis	290	522	95.79	+
LMG14477	Parakeet with sepsis	270	522	95.79	+
NCIMB8816	Italian human saliva	180	0	0	−
JCM1045	Human intestine	220	0	0	−
DSM20554	Human saliva	260	0	0	−
JCM1042	Human intestine	180	0	0	−
JCM1044	Human intestine	180	0	0	−
JCM1230	Chicken intestine	100	0	0	−

### RNA extraction and RNA-seq

Total RNA was extracted from three exponential phase cultures of *L. salivarius* UCC118 with RNAprotect (Qiagen) and the miRNeasy Mini kit (Qiagen), according to the manufacturer’s protocol with minor modifications. Mechanical lysis was performed in QIAzol with 250 mg zirconium beads (0.1 mm diameter), using a bead-beater for 30 s at 3450 oscillations min^−1^, twice. A double DNase treatment was performed on 10 µg RNA with the Turbo DNA-free kit (Ambion, Life Technologies) at 37 °C for 30 min. RNA quality was checked on an Agilent Bioanalyzer with the Agilent RNA 6000 Nano kit (Agilent Technologies). The absence of RNA degradation was checked. All the RNA Integrity Numbers (RIN) were over 8.0, validating the good quality of the RNAs. A Ribo-Zero rRNA removal kit (Bacteria) was used to deplete rRNA species. RNA quality and absence of the 16S and 23S rRNA species were checked on an Agilent Bioanalyzer with the Agilent RNA 6000 Pico kit (Agilent Technologies). RNA samples depleted in rRNA were sent to GATC Biotech for strand-specific library preparation and sequenced on an Illumina HiSeq 2000 with 100 bp single reads.

**Fig. 1.**
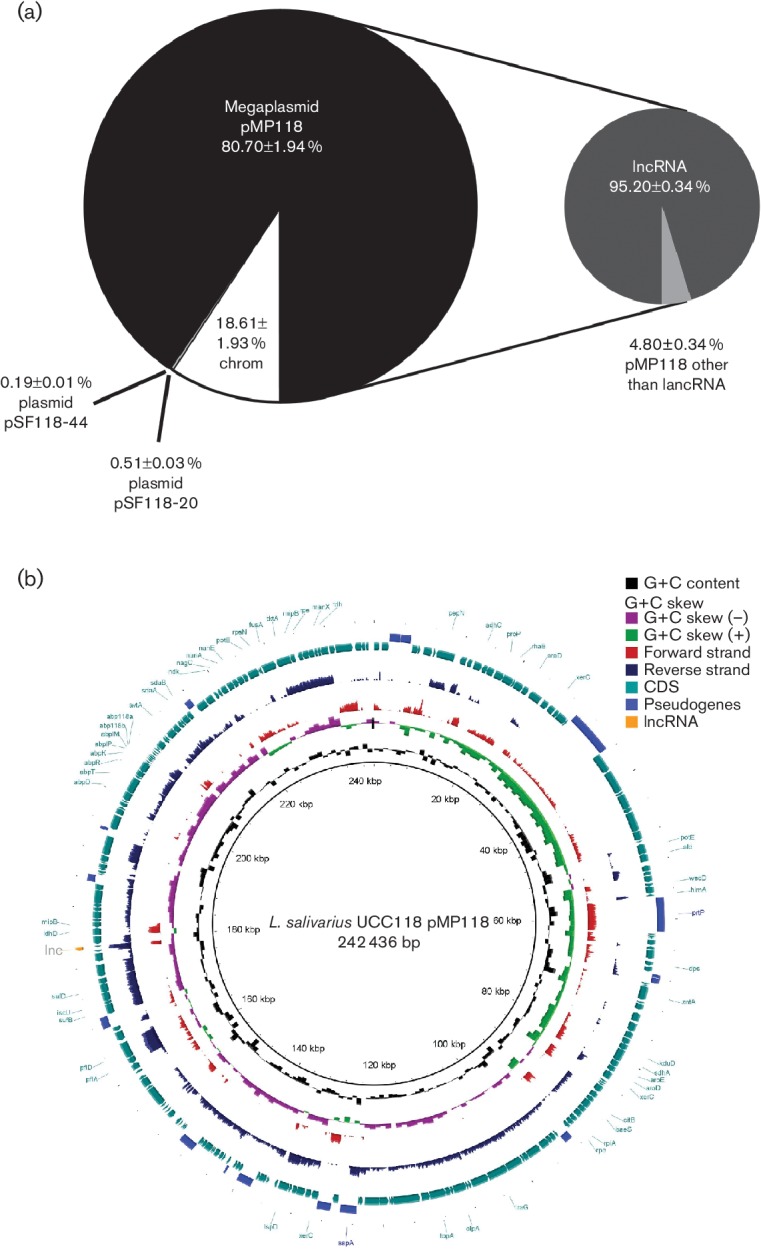

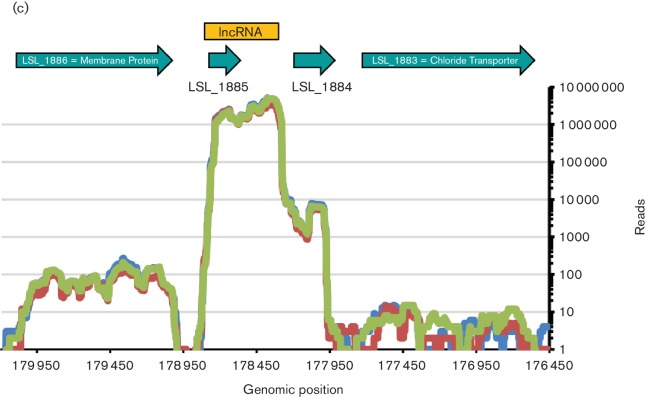
Global RNA-seq transcriptomic analysis of *L. salivarius* UCC118 in MRS. (a) Proportions of RNA-seq reads according to their location on the chromosome and the plasmids. Data presented are the mean ± sd of three replicates. (b) brig representation of the expression pattern of the *L. salivarius* UCC118 pMP118 megaplasmid. The RNA-seq reads are aligned to the reference megaplasmid pMP118 sequence of *L. salivarius* UCC118 using brig. The innermost rings show G+C content (black) and G+C skew (purple/green). The red and dark blue rings show expression patterns on a log scale (the mean of three replicates) of forward and reverse strands, respectively. The outermost rings, shown in light blue and mid blue, highlight the predicted CDS and pseudogenes of pMP118, respectively. The lncRNA is highlighted in an orange colour. (c) Zoomed in image of the expression pattern of one strand around the lncRNA genomic area. Mapped read counts of every nucleotide from positions 176 462 to 180 174 are presented for each replicate (blue, red and green lines).

Raw reads from each sample were trimmed using Trimmomatic [31] to remove the TruSeq3-SE adaptors using clip parameters 2 : 30 : 10, a sliding window of size 4 with a mean quality of 20, minimum quality of leading and trailing bases of 10, and a minimum final length of 70 nt for a read to be retained. The human genome (GRCh37) and the genome of *L. salivarius* UCC118 were combined to generate a database to which the RNA-seq reads could be aligned simultaneously. star [32] was used to align the trimmed reads to both genomes, allowing for a 0.5 ratio of mismatches to mapped length, with the remainder of parameters set to default. HTSeq [33] was used to determine the number of reads aligning to each gene, using the intersection-nonempty setting. The read numbers of each gene were expressed in RPKM (reads per kilobases per million reads) scores [34]. A custom Perl script was written to determine the number of reads that aligned to the lncRNA, the number of reads that aligned at each position along the megaplasmid pMP118 and to calculate the log_10_ of the number of reads aligning at each position. These logged counts were used as input for the blast Ring Image Generator (brig) [35], used to generate Figs 1(b) and S2(a) (available with the online Supplementary Material).

### lncRNA sequence, comparative genomics, RNA structure and quantitative PCR (qPCR) primer design

The lncRNA sequence was obtained by circular RACE experiments performed according to methods described elsewhere [36]. The circular RACE was performed on UCC118, UCC119, AH43310 and AH43324 strains, with RNAs extracted at 10 and 24 h of culture.

The lncRNA sequence was searched (blastn) against the NCBI non-redundant nucleotide database and against the 45 *L* *. salivarius* genomic sequences available in our laboratory. When a positive match for the lncRNA was obtained, the lncRNA genomic area was sequenced (Sanger sequencing method; GATC Biotech) in order to confirm the sequence. The corresponding PCRs were performed with the high-fidelity Phusion polymerase and using the primers LSL_1886_F and LSL_1883_R (Table S1).

RNA structure of the lncRNA was predicted with the lncRNA alignment in RNAalifold [37], part of the ViennaRNA Web Services. The sequences of the genes of interest from all the *L. salivarius* strains were aligned with Muscle and primers were designed within the conserved areas using the Primer3Plus Web tool (Table S1).

### RNA extraction and reverse transcription-quantitative PCR (RT-qPCR) analysis

For the lncRNA expression study with *L. salivarius* UCC118, total RNA was extracted every 2 h from 4 h (early exponential phase) to 12 h (stationary phase), and also at 24 h (late stationary phase), in quadruplicates. The RNA extraction was performed with RNAprotect (Qiagen) and the RNeasy Mini kit (Qiagen) according to the manufacturer’s protocol, with minor modifications as described above. All purified RNA samples were stored at −80 °C until further analysis. RNA quality and DNA digestion (performed on 5 µg) were carried out as described above. Lack of contamination of the RNA samples by DNA was also confirmed by qPCR. *era* primers and 0.5 ng RNA per well (the same quantity of cDNA for the RT-qPCR) were used (Table S1), with the same conditions. The absence of or low DNA contamination was confirmed with *C*_t_ values higher than 30. For the lncRNA expression study with the 27 *L* *. salivarius* strains, total RNA was extracted at exponential phase (5 h), stationary phase (10 h) and late stationary phase (24 h) as described above, in duplicates.

cDNA was synthesized using the High-Capacity cDNA Archive kit (Life Technologies). Briefly, 250 ng RNA was reverse transcribed in a final volume of 25 µl. The conditions of the reverse transcription were as follows: annealing at 25 °C for 10 min, RT at 37 °C for 2 h, and inactivation at 85 °C for 5 min. The cDNA samples were stored at −20 °C until needed for RT-qPCR.

qPCR was performed using a LightCycler 480 system (Roche). The reactions were carried out in 15 µl containing 0.25 µM each primer (Table S1), 1× SYBR Green I Master (Roche) and 5 µl of 1 : 100 diluted cDNA template. The PCR cycles consisted of one activation cycle of 5 min at 95 °C, and 40 amplification cycles of 15 s at 95 °C and 1 min at 60 °C. Each PCR product was further analysed by generating melting curves to ensure the specificity of the assay. All quantifications were performed in duplicate.

Standard curves with *L. salivarius* UCC118 genomic DNA (from 10^2^ to 10^8^ copies per well) were generated to calculate the number of copies of each gene in each sample. The expression of seven reference genes (*era*, *fusA*, g*roEL*, *gyrA*, *ileS*, *recA* and *rpoB)* was analysed to find the best normalization factor. The most stable control genes were determined with the cotton EST database. Means of the best candidate genes were also compared. qPCR data of the targeted genes were normalized by averaging the seven reference genes.

Five regions of interest in the lncRNA coding sequence were targeted: the lncRNA located on the predicted ORF (beginning) and on the tail (end) of the lncRNA sequence were studied along with *LSL_1884*, the downstream gene that encodes a predicted protein with a helix-turn-helix (HTH) motif. tmRNA, which is another lncRNA, and the 16S rRNA were also analysed.

To examine lncRNA stability, *L. salivarius* UCC118 cultures at an OD_600_ value of approximately 1.0 were treated with 400 µg rifampicin ml^−1^ and total RNA was extracted as described above just before (T0) and at 5 min intervals for 30 min after rifampicin exposure. RT-qPCR was performed as described before.

### *In vivo* expression of the lncRNA

C57Bl/6 mice were purchased from Harlan UK. Animals were kept in a conventional colony, and received food and water *ad libitum* for the duration of the experiment. Eight C57Bl/6 mice were divided into two groups. The first group received no treatment, and after sacrifice, DNA and RNA were extracted from both small and large intestine. Mice from the second group were gavaged every day with a fresh culture of *L. salivarius* UCC118 (10^10^ c.f.u. day^−1^), and DNA and RNA were extracted from both small and large intestine 2 h after the last gavage. qPCR and RT-qPCR were performed as described above. The determination of the lactobacilli level was performed as described elsewhere [38].

### Genomic DNA extraction and megaplasmid copy number determination

The genomic DNA of *L. salivarius* strains was isolated using the Qiagen DNeasy Blood and Tissue kit, according to the manufacturer’s instructions for Gram-positive bacteria. The genomic DNA was quantified using a spectrophotometer (Thermo Scientific Nanodrop 2000) and checked for integrity in a 0.8 % agarose gel.

qPCR was performed as described above on gDNA with four sets of primers targeting the chromosome (*era*, *gyrA*, *ileS*, *groEL*) and four sets of primers targeting the megaplasmid (*repA*, *pepN*, *zntA* and the lncRNA – except for the six *L* *. salivarius* strains without the lncRNA sequence in their genomes). All primer pairs gave similar efficiencies of over 90 %. The ratio of megaplasmid to chromosome was calculated by using the formula R=2^(geometric mean *C*t chromosomal genes − geometric mean *C*t megaplasmid genes)^, where *C*_t_ is the crossing threshold value.

### Creation of a lncRNA deletion mutant in *L. salivarius* UCC118

Knock-outs (deletion of the lncRNA and deletion of the lncRNA along with the *LSL_1884* gene) of the *L. salivarius* UCC118 wild-type strain (Fig. S1) were performed by a double cross-over strategy using the pORI19/pVE6007 system as described previously [3]. The bacterial strains and plasmids used for this mutant construction are presented in Table S2. Genomic DNA of *L. salivarius* UCC118 was used as a template for PCR amplification (Phusion high-fidelity DNA polymerase) of the flanking regions of the lncRNA (Table S2). The amplicons were joined by SOE-PCR (Table S2). The resulting 2 kb amplicon was digested using *BamH*I and *EcoR*I, and cloned into pORI19 digested with the same enzymes. The resulting plasmids were named pORI_19_-Δ*lncRNA* and pORI_19_-Δ*lncRNA*Δ *LSL_1884*. Deletion of the lncRNA and the lncRNA+LSL_1884 regions were further confirmed by PCR amplification using the primer pair LSL_1886_F and LSL_1883_R, which flank the lncRNA region (Table S2), and the absence of the lncRNA peak on the Bioanalyzer profiles (Fig. S1c).

### Microarray hybridization and analysis

*L. salivarius* UCC118 wild-type and both mutant strains were grown in MRS, and RNA was extracted in duplicate, as described above, in exponential and stationary phases. Labelling of cDNA with Cy3 and Cy5 dyes was carried out using a chemical labelling kit (Kreatech), following the manufacturer’s instructions. Microarray slides were hybridized for 16 h at 55 °C and scanned using an Agilent Microarray Scanner system (G2505B) with Agilent scan control software (version 7.0). Agilent feature extraction software (version 9.1) was used to process the image file and the extracted data were further processed using an in-house microarray transform platform, as previously described [2]. Genes were selected as being significantly changed in expression if their fold change in Cy3/Cy5 ratio was >3 and where the *P* value was <0.0001. Four microarray conditions were carried out in duplicate: wild-type against Δ*lncRNA* in exponential phase, wild-type against Δ*lncRNA* Δ *LSL_1884* in exponential phase, wild-type against Δ*lncRNA* in stationary phase and wild-type against Δ*lncRNA* Δ *LSL_1884* in stationary phase.

## Results

### A novel lncRNA in *L. salivarius* UCC118

RNA-seq transcriptome analysis of *L. salivarius* UCC118 was performed in triplicate (Fig. S2a), and a total of almost 42 billion reads were aligned to the *L. salivarius* UCC118 genome (Fig. S2b). Surprisingly, more than 80 % of the reads aligned to the pMP118 megaplasmid (Fig. 1a). This new unusually abundant lncRNA was sequenced by a circular RACE approach and corresponded to a 520 nt region of the megaplasmid (Fig. 1b). It represented between 74.77 and 78.80 % of the sequence reads after ribodepletion of the rRNA species (Fig. S2b), and mapped to a region derived from only 0.024 % of the length of the genome. The lncRNA physically corresponded to the predicted *LSL_1885* gene, but extended upstream and downstream, as shown in Fig. 1(c). LSL_1885 is a hypothetical protein, has no predicted function and was, by far, the highest expressed gene in the RNA-seq data (Fig. S2c). Two genes, *LSL_1883* and *LSL_1884*, are present downstream of the lncRNA coding region (Fig. 1c). The *LSL_1884* gene is annotated as a transcriptional regulator that contains a HTH motif, and the *LSL_1883* gene is predicted to encode a chloride transporter. Upstream of the lncRNA locus, the *LSL_1886* gene encodes a predicted membrane protein component of a sugar phosphotransferase system. Moreover, some RNA-seq reads also mapped on the opposite strand of the lncRNA sequence, and we hypothesized that this was an artefact due to the very high expression level of this genomic area. The occurrence of some artefactual anti-sense transcripts due to RNA-seq library preparation has already been described [39, 40].

### *L. salivarius* UCC118 lncRNA accumulates over time

**Fig. 2.**
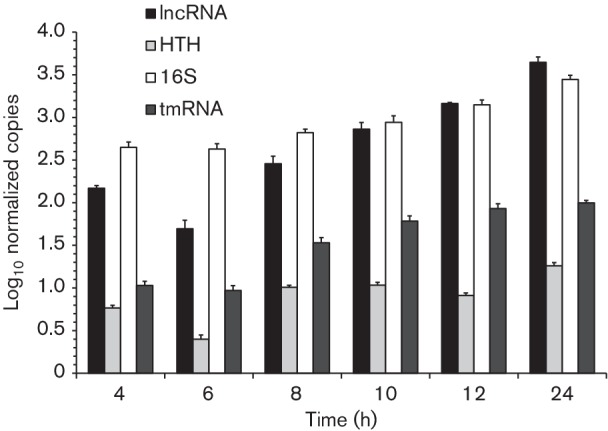
Expression level of the lncRNA during the growth of *L. salivarius* UCC118. The expression levels of lncRNA (*LSL_1885* and tail), HTH (*LSL_1884*), 16S rRNA and tmRNA were quantified by RT-qPCR after 4, 6, 8, 10, 12 and 24 h of a *L. salivarius* UCC118 culture. Data are means±sd of four independent replicates.

Total RNA was extracted at different time points in an *L. salivarius* UCC118 batch culture in order to assess the lncRNA expression level. The lncRNA was readily visible on the RNA Bioanalyzer profile, in addition to the dominant 16S and 23S rRNA peaks/bands (Fig. S3a, b). The lncRNA proportionally accumulated during *L. salivarius* UCC118 growth, and reached more than 4 % of the total (not ribodepleted) RNA by 24 h (Fig. S3b). The lncRNA expression level was confirmed by RT-qPCR, with primer pairs targeting both the predicted gene *LSL_1885* and the lncRNA tail. The tmRNA was used as a control for an accumulating ncRNA species during growth (Fig. 2), since this tmRNA is known to be a ribosome rescue system [30]. The lncRNA was confirmed to be a single transcript (Fig. S3c). The RT-qPCR analysis confirmed that the lncRNA accumulated during *L. salivarius* UCC118 growth, and by this method of measurement, it reached the abundance level of the 16S rRNA expression in stationary phase (10 and 12 h of growth), and even exceeded it 1.6-fold in late stationary phase (24 h) (Fig. 2). The transcript for the downstream transcriptional regulator *LSL_1884* did not accumulate during *L. salivarius* UCC118 growth, indicating it is part of a separate transcriptional unit.

### *L. salivarius* UCC118 lncRNA is stable and expressed *in vivo*

**Fig. 3.**
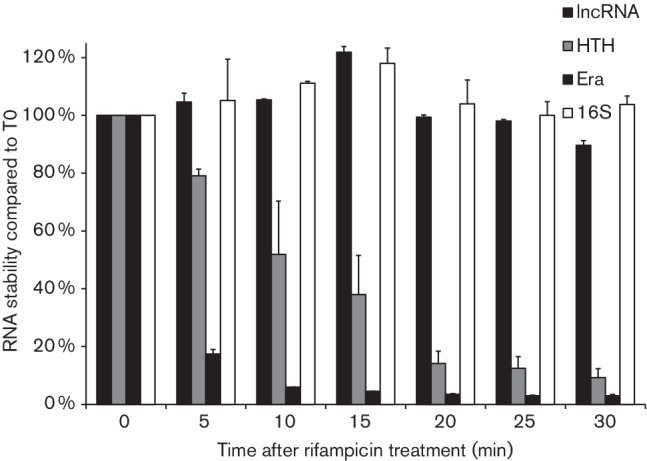
Stability over time of *L. salivarius* UCC118 lncRNA. The expression levels of lncRNA (*LSL_1885* and tail), HTH (*LSL_1884*), *era* and 16S rRNA were quantified by RT-qPCR every 5 min for 30 min after exposure of a *L. salivarius* UCC118 culture to 400 µg rifampicin ml^−1^. The expression levels were normalized and expressed as a percentage of expression before exposure to rifampicin (T0). Data are means±sd of three independent replicates.

**Fig. 4.**
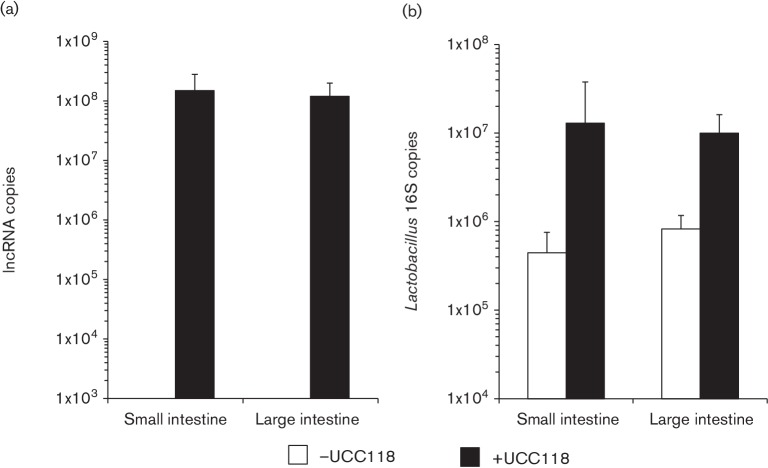
Expression of *L. salivarius* UCC118 lncRNA *in vivo*. (a) The presence of the lncRNA was quantified by RT-qPCR in both small and large colonic contents in mice (*n*=4 in each group) gavaged or not with *L. salivarius* UCC118. Data are means±sd. (b) The lactobacilli level was quantified by qPCR in both small and large colonic contents in mice (*n*=4 in each group) after genomic DNA extraction. Data are means±sd.

ncRNA is more stable than mRNA. We checked the stability of the *L. salivarius* UCC118 lncRNA by treating cells with rifampicin, which inhibits RNA polymerase. The lncRNA displayed stability similar to that of the 16S rRNA control, with only 10 % loss after 30 min for the lncRNA (Fig. 3). In contrast, the mRNA species corresponding to the *LSL_1884* gene (downstream predicted transcriptional regulator) and *era* gene (ribosome-associated GTPase, often used as a constitutively expressed housekeeping gene for RT-qPCR normalization) declined by approximately 62 and 96 % after 15 min, respectively (Fig. 3). We also confirmed the expression of the lncRNA *in vivo* during murine intestinal transit (Fig. 4a). The lncRNA was not detected in the untreated control group, whereas it was detected (more than 10^8^ copies of lncRNA) in both the small and large intestine of mice gavaged with *L. salivarius* UCC118, even though the *Lactobacillus* 16S rRNA gene was detected in both treated and untreated mice. This lncRNA presence in the gut contents of the treated group correlated with the increase of *Lactobacillus* genomic DNA in the same samples (Fig. 4b).

### lncRNA is expressed in all *L. salivarius* strains harbouring the sequence, but expression levels vary

**Fig. 5.**
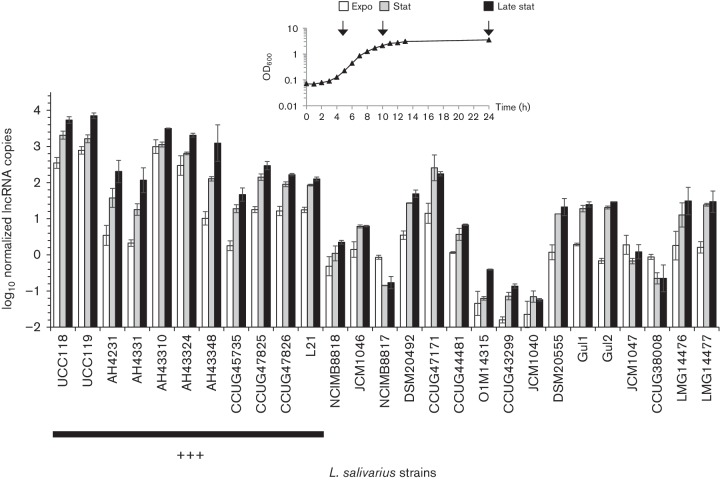
Expression levels of the lncRNA during the growth of *L. salivarius* in MRS. The expression levels of lncRNA were quantified in 27 *L* *. salivarius* strains by RT-qPCR after 5 h (exponential phase), 10 h (stationary phase) and 24 h (late stationary phase) of culture. The 11 strains sharing an identical nucleotide sequence to the *L. salivarius* UCC118 lncRNA are highlighted with +++ (see also Table 1). Data are means±sd of two independent replicates.

Comparative genomics revealed that the sequence corresponding to this newly described lncRNA was uniquely present in *L. salivarius* (i.e. absent in all other genomes in the non-redundant NCBI DNA database) and was present in 34 of the 45 *L* *. salivarius* genomes available. Twenty-seven of these strains were available as cultures in our laboratory collection, and among these, 10 harboured a lncRNA sequence identical to that of *L. salivarius* UCC118 (Table 1). The boundaries of the lncRNA were confirmed by a circular RACE approach on four strains (UCC118, UCC119, AH43310 and AH43324). The 27 lncRNA sequences were extracted from the genomes, and confirmed by Sanger sequencing, allowing a multiple alignment (Fig. S4). This alignment revealed a lncRNA size ranging from 507 to 526 nt, with fewSNPs along the alignment. Several gaps or insertions (between 6 and 13 nt) were also present in five *L* *. salivarius* strains. The multiple alignment was also used to predict the secondary structure of this lncRNA (Fig. S5). The predicted structure is a three-branched RNA with high base-pair probabilities. All 27 strains harbouring the lncRNA presented a typical bacterial growth profile (data not shown) and remained in a lag phase of up to 3 h, followed by an exponential phase of 3 to 6 h. Early stationary phase was reached from 6 to 9 h, and stationary phase was established after 9 h growth. Division rates were calculated for the 27 strains and compared to the final culture optical density values at 24 h (Fig. S6). The 11 strains sharing 100 % sequence identity for the lncRNA had lower OD_600_ values after 24 h than the other strains, except for strain CCUG45735. This was particularly true for the four strains UCC118, UCC119, AH43310 and AH43324, for which the OD_600_ values did not exceed 6.6 after 24 h. The 11 strains also showed lower growth rates. The mean generation time for the 27 *L* *. salivarius* strains was 53.6±15.9 min, which is characteristic of *Lactobacillus* growth. Total RNA was extracted for the 27 strains at exponential (5 h), stationary (10 h) and late stationary (24 h) phases. Only the RNA profiles of four strains, UCC118, UCC119, AH43310 and AH43324, showed the visible presence of the lncRNA (Fig. S5). For these four strains, the peak area for the lncRNA increased during growth showing accumulation (data not shown), as described above for *L. salivarius* UCC118 (Fig. 2). For the other strains, there was no visible peak corresponding to the lncRNA. The same four strains harboured the highest expressions of the lncRNA (Fig. 5, Table S3). For these four strains, the expression of the lncRNA was as abundant as the expression of the 16S rRNA species at the stationary and late stationary phases (Table S3). The lncRNA expression level in the other strains was lower, explaining the absence of a visible peak for the lncRNA in their RNA Bioanalyzer profiles. *L. salivarius* UCC119 had the highest expression and *L. salivarius* JCM1040 had the lowest expression of the lncRNA, with more than 45 000-fold difference in expression levels between these two strains. In addition to the high expression level of the lncRNA, this lncRNA accumulated during growth. This accumulation was observed in 24 strains, including the 11 strains sharing 100 % identity for the lncRNA sequence (Fig. 5, Table S3). Strain AH43348 showed the strongest accumulation, with a lncRNA level 100 times higher at the late stationary phase than at the exponential phase. Only three strains (NCIMB8817, JCM1047 and CCUG38008) showed a decrease in the expression of the lncRNA during growth. These three strains expressed the lncRNA at relatively low levels compared to all the others strains that accumulated the lncRNA. In addition, a strong correlation between RT-qPCR data targeting the predicted ORF (beginning) and the tail (end) of the lncRNA sequence was again observed with all the 27 *L* *. salivarius* strains (data not shown). The four *L. salivarius*, UCC118, UCC119, AH43310 and AH43324, showed a very high expression level of the lncRNA, and some specific traits such as lower OD_600_ values and higher megaplasmid copy number (Fig. S8).

The downstream *LSL_1884* gene encoding the predicted HTH protein did not follow the same expression pattern as that of the lncRNA, and there were some differences in HTH expression levels in the *L. salivarius* strains. Globally speaking, there was no accumulation of this RNA, unlike the lncRNA (Fig. S9a, Table S3). It is important to note that the four strains having the highest expression of the HTH gene were strains UCC118, UCC119, AH43310 and AH43324, which also had the highest expression of the lncRNA, and showed visible lncRNA species in their RNA profiles. The HTH gene product could have a role in the expression or the stability of the lncRNA. This might also mean that these genes share the same promoter.

The tmRNA was used in this study as a representative of the expression of a second lncRNA present in all bacteria. As predicted, the tmRNA was expressed in all *L. salivarius* strains, with a relatively high expression level, but which was lower than that of the lncRNA and 16S rRNA expression (Fig. S9b, Table S3). The expression level of the tmRNA was less variable among the *L. salivarius* strains than that of the lncRNA and HTH genes. For all strains examined, an accumulation of the tmRNA was observed. This accumulation is concordant with the literature, as this tmRNA function is relevant for stress survival, and usually increases during growth [30, 41].

### Expression level of the lncRNA correlates with megaplasmid copy number

**Fig. 6.**
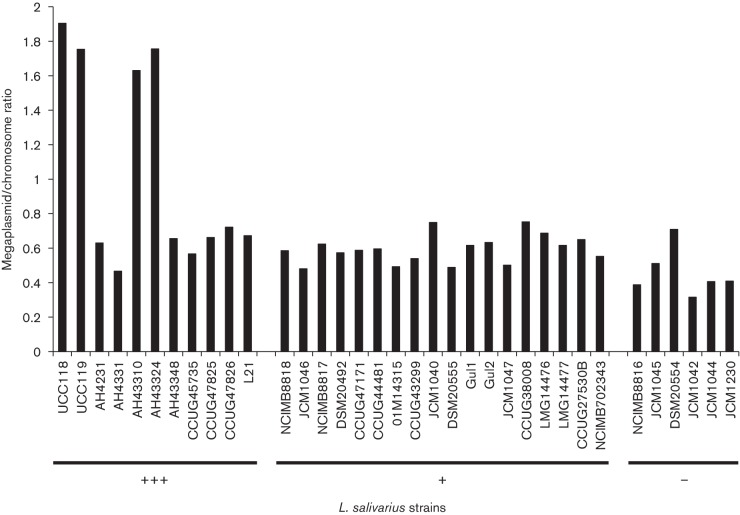
Comparison of the number of copies of chromosome and megaplasmid among 27 *L* *. salivarius* strains. The number of copies of chromosome and megaplasmid were quantified for each *L. salivarius* strain by qPCR (four genes for each genomic component). The 11 strains sharing a nucleotide sequence identical to that of *L. salivarius* UCC118 for the lncRNA are marked with +++, the strains harbouring a different sequence of the lncRNA are marked with +, and the strains without the lncRNA region in their genome are marked with −.

qPCR was performed on *L. salivarius* gDNA with three sets of primers targeting the chromosome and three sets of primers targeting the megaplasmid in order to assess the copy number ratio between these two genomic components. For most of the *L. salivarius* strains (even the strains lacking the lncRNA sequence), the ratio of megaplasmid to chromosome copy number was less than 1 (Fig. 6). Strikingly, the only strains with a higher megaplasmid copy number than chromosome equivalent were the four strains with the highest expression level of the lncRNA (Figs 6 and S7). This might indicate that the lncRNA has a role in the control of the megaplasmid number or this relationship may simply reflect gene dosage effects.

### Transcriptome analysis of a lncRNA deletion strain did not identify a distinct function

Two *L. salivarius* UCC118 knock-out mutants were created, by deletion of the lncRNA coding sequence, and by deletion of the lncRNA sequence plus the *LSL_1884* gene. The steps involved in the creation of the mutants and in the verification of the clean deletion in each construct are presented in Fig. S1. No significant change in growth rate or bacteriocin production of either *L. salivarius* UCC118 mutant was observed (data not shown). Microarray analyses of cultures in both exponential and stationary phases were performed to try to identify the function of the lncRNA. The whole transcriptomes of these two mutants were compared with the wild-type *L. salivarius* UCC118. Expression of only a few genes were up-regulated in the mutants in exponential phase, including 10 genes of the fatty acid biosynthesis operon *fab* (encompassing 13 genes) and 3 genes from the megaplasmid-encoding hypothetical proteins, two of which (LSL_1831 and LSL_1832) might be a toxin/antitoxin system (Table S4). The expression levels of *LSL_0450* and the *fabD* gene, both from the *fab* operon, were quantified by RT-qPCR in the panel of 27 *L* *. salivarius* strains and no correlation to the lncRNA expression level was observed (Table S3). In addition, a preliminary metabolomic analysis (gas chromatography-mass spectrometry) did not detect differences in the fatty-acid profile of wild-type UCC118 and the two deletion mutants (data not shown).

Expression of eighteen genes was down-regulated in the mutants compared to the wild-type. Twelve of the genes down-regulated in the mutants were harboured by the megaplasmid (Table S4). The mostly significantly repressed expression levels in the mutants were for *pflA* and *pflD* in the exponential phase. Expression of these two genes, also members of the top 10 expressed genes in *L. salivarius* UCC118 (Fig. S2c), was quantified in the 27 *L* *. salivarius* strains and again no correlation with the lncRNA expression level was observed (Table S3). As the four strains with the highest expression level of the lncRNA presented higher megaplasmid copy number than chromosome copy number, the ratio between these two genomic elements was quantified for both mutants (Table 2). The genomic deletion of the lncRNA in *L. salivarius* UCC118 resulted in a decrease of the megaplasmid/chromosome ratio. The megaplasmid copy number was still higher than the number of chromosome equivalents in both mutants (ratio >1), and was still higher than the ratio in the other *L. salivarius* strains without or with a lower expression level of lncRNA (ratio ≈ 0.5; Fig. 6).

**Table 2.** Comparison of the number of copies of the chromosome and the megaplasmid among *L. salivarius* UCC118 wild-type and lncRNA mutants

***L. salivarius* strain**	**Megaplasmid/chromosome ratio**
**Wild-type**	
UCC118	1.61
**Mutants**	
ΔlncRNA	1.16
ΔlncRNA ΔLSL_1884	1.17

## Discussion

Data from the increasing number of bacterial genomes and NGS transcriptomic studies have indicated that ncRNAs play an important role in bacteria, although notably most studies have been carried out on pathogenic bacteria [10, 42]. RNA-seq studies are additionally informative, because they provide enhancement of the accuracy of existing genome annotations, for example in *Bacillus anthracis* [43]. A few RNA-seq studies have been recently described in *Lactobacillus* species [44–49]. The study by Zheng *et al.* on *Lactobacillus delbrueckii* subsp. *bulgaricus* highlighted the potential application of ncRNA for regulating gene expression in lactic acid bacteria [44]. A comparative genomics study also revealed multiple potential ncRNAs in lactobacilli [50], but the phenomenon is still under-examined in this group of bacteria. Our intended RNA-seq study of ncRNA species across the whole genome of the commensal bacterium *L. salivarius* UCC118 was unexpectedly re-directed into trying to understand the role of the single large ncRNA that dominated the transcriptome.

The existence of this new lncRNA, specific to the *L. salivarius* species, was supported by both RNA-seq and RT-qPCR data, which are also in accordance with previous microarray experiments in which *LSL_1885* and 16S rRNA raw spot intensities were very high and of similar intensity [2]. It is not clear whether the predicted *LSL_1885* gene is actually translated or whether it is a false positive of the gene prediction process. That *LSL_1885* might not be a real gene is supported by the fact that some *L. salivarius* strains (NIAS840, ACS-116-V-Col5a, DSM20555^T^, Ren) harbouring an identical or close sequence to the lncRNA of *L. salivarius* UCC118 annotated a shorter gene homologous to *LSL_1885*, or did not present a predicted gene in this genomic area [51–54]. This is one location where reannotation of *L. salivarius* genomes may be improved. There is also evidence of expression of loci that have been annotated as pseudogenes. It is possible that these were misannotated or misassembled previously, for which the RNA-seq data presented here could assist in a reassembly. However, it is also possible that these pseudogenes are real and recently lost, and cannot be translated, but still maintain their former expression. Further analysis is required to determine the true state of such genes.

lncRNA species in prokaryotes can be large, ranging from 700 to 3500 nt, but these are typically anti-sense species that affect transcription, RNA stability or translation (reviewed in [17]). *LSL_1884/LSL_1885* does not directly overlap any predicted genes or display sequence homology to any other genomic regions. In contrast to convergent untranslated overlapping RNAs (CutoRNAs) of *Streptomyces* [20], the lncRNA in *L. salivarius* does not overlap by 3′ overhang a downstream gene. Furthermore, we would have expected to detect effects on transcription or RNA stability of putative target genes for the *L. salivarius* lncRNA when we compared the transcriptome of wild-type and *LSL_1884/LSL_1885* deletion mutants, but the effects of deleting the lncRNA were inconclusive. Expression of the 11 gene operon from *LSL_0449* to *LSL_0459*, dominated by fatty acid biosynthesis genes, was significantly and uniformly up-regulated in both deletion mutants. However, a preliminary metabolomic analysis (gas chromatography-mass spectrometry) did not detect any differences in the total cellular fatty-acid profile of wild-type UCC118 cultures and the two deletion mutants (data not shown). It is still unclear why the lncRNA is expressed in such high abundance but does not seem to play a major role in the strain growth or metabolism, at least according to the approaches we took, including two knock-out constructions. A full proteomic and metabolomic analysis of wild-type and deletion mutants would be required to seek to identify phenotypes dependent on the lncRNA.

The expression of several coding stretches on the megaplasmid was significantly reduced by lncRNA deletion, including two genes involved in formate metabolism (Table S4). Pyruvate formate lyase is an oxygen-sensitive enzyme that regulates anaerobic fermentation by controlling acetyl coenzyme A production [55]. It is not obvious why *L. salivarius*, or more correctly only a sub-set of strains of *L. salivarius*, might have evolved a complex ncRNA mechanism for controlling such a universal metabolic feature. Furthermore, the growth rate of the wild-type strain was essentially identical to either of the lncRNA deletion mutants when grown aerobically or microaerobically (5 % CO_2_; data not shown).

The *L. salivarius* lncRNA species is very stable. A prior example of a well characterized, unusually stable *Lactobacillus* mRNA is that encoding the surface layer protein on *Lactobacillus brevis* cells [56], which has an unusually long half-life of 14 min. We did not measure a lncRNA half-life in rifampicin-treated cells in which the lncRNA suffered only 10 % loss after 30 min. RNA molecules, especially non-coding long species, may be stabilized by secondary structure [57]. The *L. salivarius* lncRNA is smaller than the *L. plantarum* supermotifs [26] that are stable, transcribed, intergenic RNA species with a cruciform structure, which the *L. salivarius* lncRNA is not predicted to assume. The reason for the unusual stability of the *L. salivarius* lncRNA is currently unclear.

The presence of megaplasmids is a distinguishing and unifying feature of *L. salivarius*, and these plasmids range in size from 100 kb to approximately 400 kb, in linear or circular forms [6]. Sequence analysis in our laboratory of 45 *L* *. salivarius* genomes confirmed that none of these plasmids contain unique copies of any genes annotated as essential for cell survival [5]. The fact that the lncRNA-encoding sequence is borne on the megaplasmid is probably significant. However, it cannot be argued that the lncRNA is, for example, essential for megaplasmid maintenance or replication, since not all strains with megaplasmids harbour the coding sequence. The correlation of expression levels of lncRNA with megaplasmid copy number appears not to be exclusively a gene-dosage effect, because deleting the coding sequence for the lncRNA significantly reduced megaplasmid copy number. However, this may be an indirect effect, whereby lncRNA modulates the expression or efficiency of an unknown plasmid or chromosomally encoded function that impacts on plasmid replication. This has already been described with asRNAs, some of which regulate plasmid copy number in bacteria [58, 59]. A large study from Weinberg *et al*. also mentioned ncRNA with a putative role in plasmid copy regulation in *Lactobacillus* sp. [50].

asRNA molecules are recognized components of toxin/antitoxin systems [60], whereby they act to inhibit translation of the toxin mRNA (type I antitoxin) or to directly inhibit the activity of their cognate protein toxin by binding to it (type III antitoxin). Deletion of *LSL_1885/LSL_1884* led to up-regulation of two genes on the megaplasmid, *LSL_1831* and *LSL_1832*, that are annotated as hypothetical proteins, but which have low-level homology to toxin/antitoxin proteins. LSL_1831 may be a PIN-domain ribonuclease and LSL_1832 contains a domain found in type II antitoxins. The combination in the lncRNA deletion mutant of reduced plasmid copy number, and increased expression of a candidate plasmid addiction system, suggests that the lncRNA might be involved in maintaining megaplasmids in *L. salivarius*, but this requires experimental investigation.

The lncRNA is very stable, and the RNA species is also produced *in vivo* during murine intestinal transit. A combination of constitutive expression and RNA stability would explain the observed accumulation of the lncRNA species during growth. Preliminary experiments using a reporter gene mapped the promoter to between −225 and −450 nt, and the expression level was comparable to that of the *cysK* promoter (data not shown), which we previously ranked among the top 3 % of highly expressed genes in *L. salivarius* [61]. Further analysis of the promoter for the lncRNA species may enable its exploitation for the sparsely resourced genetic tool-box for lactobacilli, including for use in driving selectable-marker expression in plasmid construction, or for expressing heterologous genes constitutively.

This study describes for the first time, to the best of our knowledge, a lncRNA with unusually high abundance levels in *L. salivarius*. This lncRNA presents distinctive and unique properties, which suggests potential for basic and applied scientific exploitation.

## Data bibliography

1. Cousin FJ, Lynch DB, Chuat V, Bourin MJB, Casey PG *et al*. Sequence Read Archive PRJNA355319 https://www.ncbi.nlm.nih.gov/bioproject/PRJNA355319 (2017).
2. Cousin FJ, Lynch DB, Chuat V, Bourin MJB, Casey PG *et al*. GenBank MF114321 (2017).
3. Cousin FJ, Lynch DB, Chuat V, Bourin MJB, Casey PG *et al*. GenBank MF114322 (2017).
4. Cousin FJ, Lynch DB, Chuat V, Bourin MJB, Casey PG *et al*. GenBank MF114323 (2017).
5. Cousin FJ, Lynch DB, Chuat V, Bourin MJB, Casey PG *et al*. GenBank MF114324 (2017).
6. Cousin FJ, Lynch DB, Chuat V, Bourin MJB, Casey PG *et al*. GenBank MF114325 (2017).
7. Cousin FJ, Lynch DB, Chuat V, Bourin MJB, Casey PG *et al*. GenBank MF114326 (2017).
8. Cousin FJ, Lynch DB, Chuat V, Bourin MJB, Casey PG *et al*. GenBank MF114327 (2017).
9. Cousin FJ, Lynch DB, Chuat V, Bourin MJB, Casey PG *et al*. GenBank MF114328 (2017).
10. Cousin FJ, Lynch DB, Chuat V, Bourin MJB, Casey PG *et al*. GenBank MF114329 (2017).
11. Cousin FJ, Lynch DB, Chuat V, Bourin MJB, Casey PG *et al*. GenBank MF114330 (2017).
12. Cousin FJ, Lynch DB, Chuat V, Bourin MJB, Casey PG *et al*. GenBank MF114331 (2017).
13. Cousin FJ, Lynch DB, Chuat V, Bourin MJB, Casey PG *et al*. GenBank MF114332 (2017).
14. Cousin FJ, Lynch DB, Chuat V, Bourin MJB, Casey PG *et al*. GenBank MF114333 (2017).
15. Cousin FJ, Lynch DB, Chuat V, Bourin MJB, Casey PG *et al*. GenBank MF114334 (2017).
16. Cousin FJ, Lynch DB, Chuat V, Bourin MJB, Casey PG *et al*. GenBank MF114335 (2017).
17. Cousin FJ, Lynch DB, Chuat V, Bourin MJB, Casey PG *et al*. GenBank MF114336 (2017).
18. Cousin FJ, Lynch DB, Chuat V, Bourin MJB, Casey PG *et al*. GenBank MF114337 (2017).
19. Cousin FJ, Lynch DB, Chuat V, Bourin MJB, Casey PG *et al*. GenBank MF114338 (2017).
20. Cousin FJ, Lynch DB, Chuat V, Bourin MJB, Casey PG *et al*. GenBank MF114339 (2017).
21. Cousin FJ, Lynch DB, Chuat V, Bourin MJB, Casey PG *et al*. GenBank MF114340 (2017).
22. Cousin FJ, Lynch DB, Chuat V, Bourin MJB, Casey PG *et al*. GenBank MF114341 (2017).
23. Cousin FJ, Lynch DB, Chuat V, Bourin MJB, Casey PG *et al*. GenBank MF114342 (2017).
24. Cousin FJ, Lynch DB, Chuat V, Bourin MJB, Casey PG *et al*. GenBank MF114343 (2017).
25. Cousin FJ, Lynch DB, Chuat V, Bourin MJB, Casey PG *et al*. GenBank MF114344 (2017).
26. Cousin FJ, Lynch DB, Chuat V, Bourin MJB, Casey PG *et al*. GenBank MF114345 (2017).
27. Cousin FJ, Lynch DB, Chuat V, Bourin MJB, Casey PG *et al*. GenBank MF114346 (2017).
28. Cousin FJ, Lynch DB, Chuat V, Bourin MJB, Casey PG *et al*. GenBank MF114347 (2017).
29. Cousin FJ, Lynch DB, Chuat V, Bourin MJB, Casey PG *et al*. Gene Expression Omnibus GSE92837 https://www.ncbi.nlm.nih.gov/geo/query/acc.cgi?token=kzynyucqxvaldgj&acc=GSE92837 (2017).

## Supplementary Data

2479 Supplementary File 1
