# Supplementary material for: A long and abundant non-coding RNA in Lactobacillus salivarius
Source: Microb Genom. 2017 Jul 17;3(9):e000126. doi: 10.1099/mgen.0.000126 (PMC5643018; doi:10.1099/mgen.0.000126)
Supplement: Supplementary File 1 [file mgen-3-126-s001.pdf]

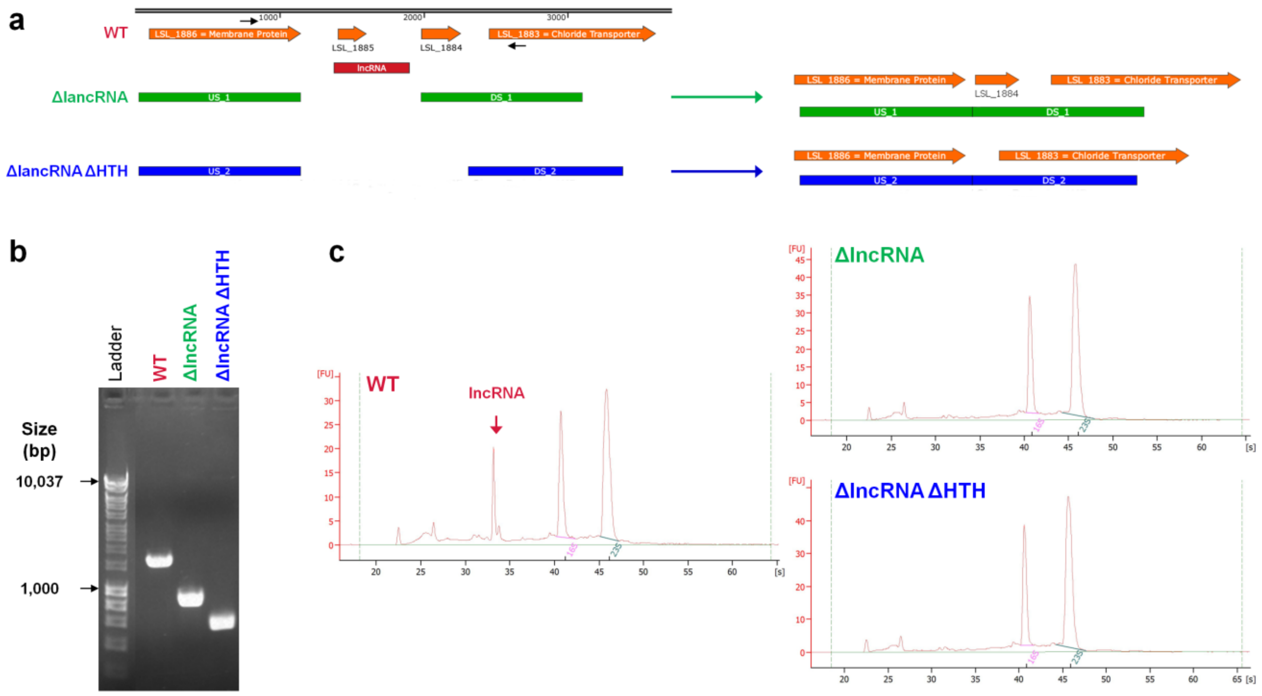

FIGURE S1: Construction and confirmation of the lncRNA deletion in *L. salivarius* UCC118 mutants. **(A)** Schematic representation of the Δ*lncRNA* and Δ*lncRNA*Δ*HTH* mutant constructions using the pORI19-pVE6007 system according to the materials and methods section. **(B)** Confirmation of the lncRNA genomic area deletions by PCR. The primer specific sites are indicated by arrows on panel A. The expected lengths of genomic fragments amplified by this primer set are 1,727 bp, 947 bp and 639 bp for *L. salivarius* UCC118 WT, Δ*lncRNA* mutant and Δ*lncRNA*Δ*HTH* mutant, respectively. **(C)** Confirmation of the absence of lncRNA expression in both mutants of *L. salivarius* UCC118. RNA electropherograms of the *L. salivarius* UCC118 WT and mutants obtained from the Agilent 2100 Bioanalyzer System.

a

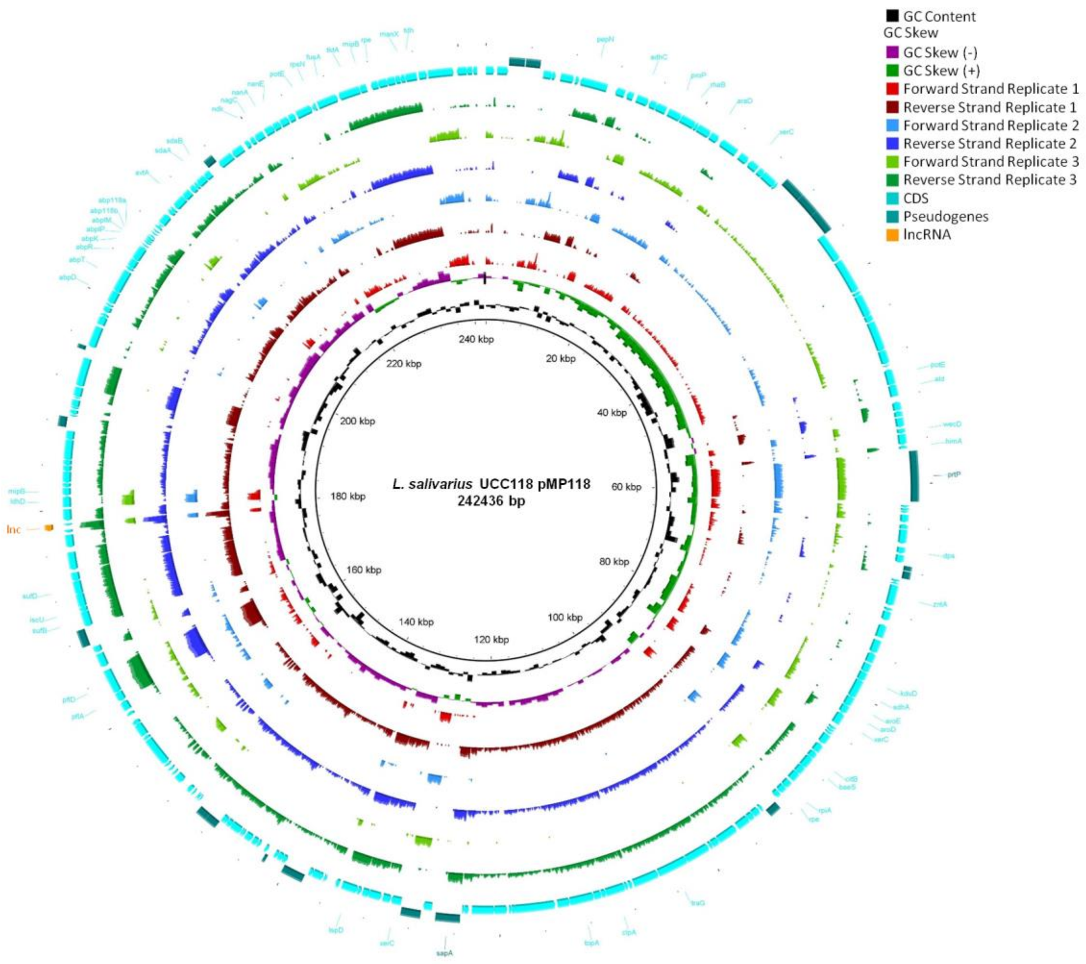

b *Read counts of the RNA-seq study of L. salivarius UCC118*

	Replicate 1		Replicate 2		Replicate 3	
**Target**	Aligned reads	Proportion	Aligned reads	Proportion	Aligned reads	Proportion
Chromosome	3,186,857	20.43%	1,998,281	16.58%	2,684,851	18.81%
Plasmid pSF118-20	83,279	0.53%	60,870	0.51%	69,028	0.48%
Plasmid pSF118-44	30,574	0.20%	23,485	0.19%	25,547	0.18%
Megaplasmid pMP118	12,302,009	78.85%	9,966,148	82.71%	11,494,398	80.53%
lncRNA	11,665,902	74.77%	9,494,604	78.80%	10,976,910	76.90%
Total	15,602,719		12,048,784		14,273,824	

c *Top 10 expressed genes from the RNA-seq transcriptomic study of L. salivarius UCC118*

**Locus**	**Name**	**Predicted function**	**Genomic position**	**Expression level[a]**
LSL_1885	-	Hypothetical protein LSL_1885	Megaplasmid pMP118	1206.34 ± 154.67
LSL_1873	*pflD*	Formate acetyltransferase	Megaplasmid pMP118	8.62 ± 0.67
LSL_1976	-	Hypothetical protein pSF118-20_17	Plasmid pSF118-20	8.35 ± 0.39
LSL_0642	*tufB*	Elongation factor Tu	Chromosome	7.52 ± 0.33
LSL_1872	*pflA*	Pyruvate formate-lyase activating enzyme	Megaplasmid pMP118	6.76 ± 0.22
LSL_1982	-	Hypothetical protein pSF118-20_23	Plasmid pSF118-20	5.62 ± 0.65
LSL_1166	*gapA*	Glyceraldehyde 3-phosphate dehydrogenase	Chromosome	3.23 ± 0.17
LSL_1738	*rpmH*	50S ribosomal protein L34	Chromosome	3.21 ± 0.24
LSL_0406	*fba*	Fructose-bisphosphate aldolase	Chromosome	2.99 ± 0.14
LSL_1884	-	Hypothetical protein LSL_1884	Megaplasmid pMP118	2.86 ± 0.08

FIGURE S2: RNA-seq data analysis of *L. salivarius* UCC118 transcriptome. (A) BLAST Ring Image Generator representation of the global RNA-seq expression pattern of the pMP118 megaplasmid of *L. salivarius* UCC118 in the independent replicates. The RNA-seq reads are aligned to the reference megaplasmid pMP118 sequence of *L. salivarius* UCC118 for each replicate. The innermost rings show GC content (black) and GC skew (purple/green). The red, blue and green rings show expression patterns for each replicate on a log scale of forward (light colors) and reverse (dark colors) strands, respectively. The outermost rings, shown in turquoise and teal, highlight the predicted CDS and pseudogenes of pMP118. The lncRNA is highlighted in an orange color. **(B)** Read counts of the RNA-seq study of *L. salivarius* UCC118 for each replicate. **(C)** Top 10 expressed genes from the RNA-seq transcriptomic study of *L. salivarius* UCC118. [a] Expression level is expressed as RPKM scores (according to the material and method section), mean of 3 replicates ± standard deviation.

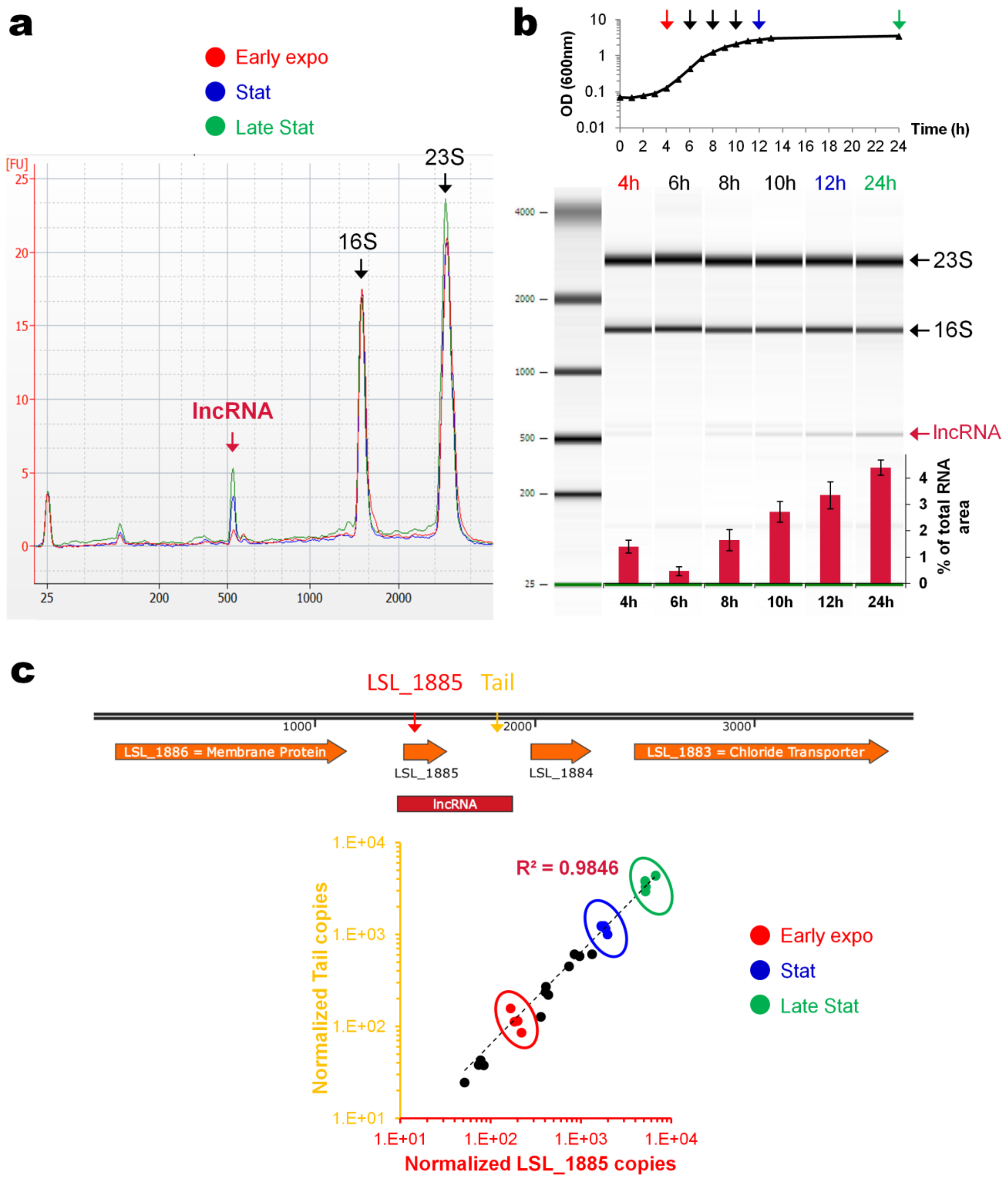

FIGURE S3: RNA profiles during the growth of *L. salivarius* UCC118 (A) RNA electropherograms of *L. salivarius* UCC118 at early exponential phase (red, 4h), stationary phase (blue, 12h) and late stationary phase (green, 24h) visualized on an Agilent 2100 Bioanalyzer System. The lncRNA specific peak is highlighted in dark pink. **(B)** Gel electrophoresis patterns during the growth of *L. salivarius* UCC118 obtained with the Agilent 2100 Bioanalyzer System. The lncRNA specific peak is highlighted in dark pink. The bar chart shows the lncRNA proportion in the total RNA for each timepoint. Data are means ± SD of 3 independent replicates. **(C)** Correlation between RT-qPCR data targeting the beginning and tail of the predicted ORF of the lncRNA sequence. RNA was extracted during the growth of *L. salivarius* UCC118 at 4h, 6h, 8h, 10h, 12h and 24h in quadruplicates.

```
UCC118         GTACCGATGAAGCTAGTGGATGAGGTGTGACAAGCCGCCTAGCCATACGACTCTTAATAA  60
UCC119         GTACCGATGAAGCTAGTGGATGAGGTGTGACAAGCCGCCTAGCCATACGACTCTTAATAA  60
AH4231         GTACCGATGAAGCTAGTGGATGAGGTGTGACAAGCCGCCTAGCCATACGACTCTTAATAA  60
AH4331         GTACCGATGAAGCTAGTGGATGAGGTGTGACAAGCCGCCTAGCCATACGACTCTTAATAA  60
AH43310        GTACCGATGAAGCTAGTGGATGAGGTGTGACAAGCCGCCTAGCCATACGACTCTTAATAA  60
AH43324        GTACCGATGAAGCTAGTGGATGAGGTGTGACAAGCCGCCTAGCCATACGACTCTTAATAA  60
AH43348        GTACCGATGAAGCTAGTGGATGAGGTGTGACAAGCCGCCTAGCCATACGACTCTTAATAA  60
CCUG45735      GTACCGATGAAGCTAGTGGATGAGGTGTGACAAGCCGCCTAGCCATACGACTCTTAATAA  60
CCUG47825      GTACCGATGAAGCTAGTGGATGAGGTGTGACAAGCCGCCTAGCCATACGACTCTTAATAA  60
CCUG47826      GTACCGATGAAGCTAGTGGATGAGGTGTGACAAGCCGCCTAGCCATACGACTCTTAATAA  60
L21            GTACCGATGAAGCTAGTGGATGAGGTGTGACAAGCCGCCTAGCCATACGACTCTTAATAA  60
NCIMB8818      GTACCGATGAAGCTAGTGGATGAGGTGTGACAAGCCGCCTAGCCATACGTCTCTTAATAA  60
JCM1046        GTACCGATGAAGCTAGTGGATGAGGTGTGACAAGCCGCCTAGCCATACGTCTCTTAATAA  60
NCIMB8817      GTACCGATGAAGCTAGTGGATGAGGTGTGACAAGCCGCCTAGCCATACGTCTCTTAATAA  60
DSM20492       ATACCGATGAAGCTAGTGGATAAGGTGTGACAAGTCGCCTAGCCATACGTCTCTTAATAA  60
CCUG47171      GTACCGATGAAGCTAGTGGATGAGGTGTGACAAGCCGCCTAGCCATACGTCTCTTAATAA  60
CCUG44481      GTACCGATGAAGCTAGTGGATAAGGTGTGACAAGCCGCCTAGCCATACGTCTCTTAATAA  60
01M14315       GTACCGATGAAGCTAGTGGATAAGGTGTGACAAGCCGCCTAGCCATACGTCTCTTAATAA  60
CCUG43299      GTACCGATGAAGCTAGTGGATAAGGTGTGACAAGCCGCCTAGCCATACGTCTCTTAATAA  60
JCM1040        GTACCGATGAAGCTAGTGGATAAGGTGTGACAAGCCGCCTAGCCATACGTCTCTTAATAA  60
DSM20555T      GTACCGATGAAGCTAGTGGATAAGGTGTGACAAGCCGCCTAGCCATACGTCTCTTAATAA  60
Gul1           GTACCGATGAAGCTAGTGGATAAGGTGTGACAAGCCGCCTAGCCATACGTCTCTTAATAA  60
Gul2           GTACCGATGAAGCTAGTGGATAAGGTGTGACAAGCCGCCTAGCCATACGTCTCTTAATAA  60
JCM1047        GTACCGATGAAGCTAGTGGATAAGGTGTGACAAGCCGCCTAGCCATACGTCTCTTAATAA  60
CCUG38008      GTACCGATGAAGCTAGTGGATGAGGTGTGACAAGCCGCCTAGCCATACGTCTCTTAATAA  60
LMG14476       GTACCGATGAAGCTAGTGGATAAGGTGTGACAAGCCGCCTAGCCATACGTCTCTTAATAA  60
LMG14477       GTACCGATGAAGCTAGTGGATAAGGTGTGACAAGCCGCCTAGCCATACGTCTCTTAATAA  60
               .********************.************ **************:**********

UCC118         CTACTATGACGAAATATACGGATACGTTTATTTTTTCTAATTTCCACTTGGGTAGTACTA  120
UCC119         CTACTATGACGAAATATACGGATACGTTTATTTTTTCTAATTTCCACTTGGGTAGTACTA  120
AH4231         CTACTATGACGAAATATACGGATACGTTTATTTTTTCTAATTTCCACTTGGGTAGTACTA  120
AH4331         CTACTATGACGAAATATACGGATACGTTTATTTTTTCTAATTTCCACTTGGGTAGTACTA  120
AH43310        CTACTATGACGAAATATACGGATACGTTTATTTTTTCTAATTTCCACTTGGGTAGTACTA  120
AH43324        CTACTATGACGAAATATACGGATACGTTTATTTTTTCTAATTTCCACTTGGGTAGTACTA  120
AH43348        CTACTATGACGAAATATACGGATACGTTTATTTTTTCTAATTTCCACTTGGGTAGTACTA  120
CCUG45735      CTACTATGACGAAATATACGGATACGTTTATTTTTTCTAATTTCCACTTGGGTAGTACTA  120
CCUG47825      CTACTATGACGAAATATACGGATACGTTTATTTTTTCTAATTTCCACTTGGGTAGTACTA  120
CCUG47826      CTACTATGACGAAATATACGGATACGTTTATTTTTTCTAATTTCCACTTGGGTAGTACTA  120
L21            CTACTATGACGAAATATACGGATACGTTTATTTTTTCTAATTTCCACTTGGGTAGTACTA  120
NCIMB8818      CTACTATGACGAAATATACGGATACGTTTATTTTTTCTAATTTCCACTTGGGTAGTACTA  120
JCM1046        CTACTATGACGAAATATACGGATACGTTTATTTTTTCTAATTTCCACTTGGGTAGTACTA  120
NCIMB8817      CTACTATGACGAAATATACGGATACGTTTATTTTTTCTAATTTCCACTTGGGTAGTACTA  120
DSM20492       CTACTATGACGAAATATACGGATACGTTTATTTTTTCTAATTTCCACTTGGGTAGTACTA  120
CCUG47171      CTACTATGACGAAATATACGGATACGTTTATTTTTTCTAATTTCCACTTGGGTAGTACTA  120
CCUG44481      CTACTATGACGAAATATACGGATACGTTTATTTTTTCTAATTTCCACTTAGGTAATACTA  120
01M14315       CTACTATGACGAAATATACGGATACGTTTATTTTTTCTAATTTCCACTTGGGTAGTACTA  120
CCUG43299      CTACTATGACGAAATATACGGATACGTTTATTTTTTCTAATTTCCACTTGGGTAGTACTA  120
JCM1040        CTACTATGACGAAATATACGGATACGTTTATTTTTTCTAATTTCCACTTGGGTAGTACTA  120
DSM20555T      CTACTATGACGAAATATACGGATACGTTTATTTTTTCTAATTTCCACTTGGGTAGTACTA  120
Gul1           CTACTATGACGAAATATACGGATACGTTTATTTTTTCTAATTTCCACTTGGGTAGTACTA  120
Gul2           CTACTATGACGAAATATACGGATACGTTTATTTTTTCTAATTTCCACTTGGGTAGTACTA  120
JCM1047        CTACTATGACGAAATATACGGATACGTTTATTTTTTCTAATTTCCACTTGGGTAGTACTA  120
CCUG38008      CTACTATGACGAAATATACGGATACGTTTATTTTTTCTAAT-------------GTACTA  107
LMG14476       CTACTATGACGAAATATACGGATACGTTTATTTTTTCTAATTTCCACTTGGGTAGTACTA  120
LMG14477       CTACTATGACGAAATATACGGATACGTTTATTTTTTCTAATTTCCACTTGGGTAGTACTA  120
               *****************************************             .*****
```

```
UCC118          ATTGGGAGCAACGAAA-AAAGTTGTGTAGAGAGAAGCAAGGGGACTCATATCTGAAACTA  179
UCC119          ATTGGGAGCAACGAAA-AAAGTTGTGTAGAGAGAAGCAAGGGGACTCATATCTGAAACTA  179
AH4231          ATTGGGAGCAACGAAA-AAAGTTGTGTAGAGAGAAGCAAGGGGACTCATATCTGAAACTA  179
AH4331          ATTGGGAGCAACGAAA-AAAGTTGTGTAGAGAGAAGCAAGGGGACTCATATCTGAAACTA  179
AH43310         ATTGGGAGCAACGAAA-AAAGTTGTGTAGAGAGAAGCAAGGGGACTCATATCTGAAACTA  179
AH43324         ATTGGGAGCAACGAAA-AAAGTTGTGTAGAGAGAAGCAAGGGGACTCATATCTGAAACTA  179
AH43348         ATTGGGAGCAACGAAA-AAAGTTGTGTAGAGAGAAGCAAGGGGACTCATATCTGAAACTA  179
CCUG45735       ATTGGGAGCAACGAAA-AAAGTTGTGTAGAGAGAAGCAAGGGGACTCATATCTGAAACTA  179
CCUG47825       ATTGGGAGCAACGAAA-AAAGTTGTGTAGAGAGAAGCAAGGGGACTCATATCTGAAACTA  179
CCUG47826       ATTGGGAGCAACGAAA-AAAGTTGTGTAGAGAGAAGCAAGGGGACTCATATCTGAAACTA  179
L21             ATTGGGAGCAACGAAA-AAAGTTGTGTAGAGAGAAGCAAGGGGACTCATATCTGAAACTA  179
NCIMB8818       ATTGGGAGCAACGAAA-AAAGTTGTGTAGAGAGAAGCAAGGGGACTCATATCTGAAACTA  179
JCM1046         ATTGGGAGCAACGAAAAAAAGTTGTGTAGAGAGAAGCAAGGGGACTCATATCTGAAACTA  180
NCIMB8817       ATTGGGAGAAACGAAAAAAAGTTGTGTAGAGAGAAGCAAGGGGACTCATATCTGAAACTA  180
DSM20492        ATTGGGAGCAACGAAAAAAAGTTGTGTAGAGAGAAGCAAGGGGACTCATATCTGAAACTA  180
CCUG47171       ATTGGGAGCAACGAAAAAAAGTTGTGTAGAGAGAAGCAAGGGGACTCATATCTGAAACTA  180
CCUG44481       ATTGGGAGCAACGAAA-AAAGTTGTGTAGAGAGAAGCAAGGGGACTCATATCTGAAACTA  179
01M14315        ATTGGGAGCAACGAAAAAAAGTTGTGTAGAGAGAAGCAAGGGGACTCATATCTGAAACTA  180
CCUG43299       ATTGGGAGCAACGAAAAAAAGTTGTGTAGAGAGAAGCAAGGGGACTCATATCTGAAACTA  180
JCM1040         ATTGGGAGCAACGAAAAAAAGTTGTGTAGAGAGAAGCAAGGGGACTCATATCTGAAACTA  180
DSM20555T       ATTGGGAGCAACGAAAAAAAGTTGTGTAGAGAGAAGCAAGGGGACTCATATCTGAAACTA  180
Gul1            ATTGGGAGCAACGAAAAAAAGTTGTGTAGAGAGAAGCAAGGGGACTCATATCTGAAACTA  180
Gul2            ATTGGGAGCAACGAAAAAAAGTTGTGTAGAGAGAAGCAAGGGGACTCATATCTGAAACTA  180
JCM1047         ATTGGGAGCAACGAAAAAAAGTTGTGTAGAGAGAAGCAAGGGGACTCATATCTGAAACTA  180
CCUG38008       ATTGGGAGCAACGAAAAAAAGTTGTGTAGAGAGAAGCAAGGGGACTCATATCTGAAACTA  167
LMG14476        ATTGGGAGCAACGAAAAAAAGTTGTGTAGAGAGAAGCAAGGGGACTCGTATCTGAAACTA  180
LMG14477        ATTGGGAGCAACGAAAAAAAGTTGTGTAGAGAGAAGCAAGGGGACTCGTATCTGAAACTA  180
                ********.******* *****************************.************

UCC118          AGACACTTGCCGTCAGGGATTCGCAATATGCCATCTTCAGTTAATTAAGGCTGATTACCT  239
UCC119          AGACACTTGCCGTCAGGGATTCGCAATATGCCATCTTCAGTTAATTAAGGCTGATTACCT  239
AH4231          AGACACTTGCCGTCAGGGATTCGCAATATGCCATCTTCAGTTAATTAAGGCTGATTACCT  239
AH4331          AGACACTTGCCGTCAGGGATTCGCAATATGCCATCTTCAGTTAATTAAGGCTGATTACCT  239
AH43310         AGACACTTGCCGTCAGGGATTCGCAATATGCCATCTTCAGTTAATTAAGGCTGATTACCT  239
AH43324         AGACACTTGCCGTCAGGGATTCGCAATATGCCATCTTCAGTTAATTAAGGCTGATTACCT  239
AH43348         AGACACTTGCCGTCAGGGATTCGCAATATGCCATCTTCAGTTAATTAAGGCTGATTACCT  239
CCUG45735       AGACACTTGCCGTCAGGGATTCGCAATATGCCATCTTCAGTTAATTAAGGCTGATTACCT  239
CCUG47825       AGACACTTGCCGTCAGGGATTCGCAATATGCCATCTTCAGTTAATTAAGGCTGATTACCT  239
CCUG47826       AGACACTTGCCGTCAGGGATTCGCAATATGCCATCTTCAGTTAATTAAGGCTGATTACCT  239
L21             AGACACTTGCCGTCAGGGATTCGCAATATGCCATCTTCAGTTAATTAAGGCTGATTACCT  239
NCIMB8818       AGACACTTGCCGTCAGGGATTCGCAATATGCCATCTTCAGTTAATTAAGGCTGATTACCT  239
JCM1046         AGACACTTGCCGTCAGGGATTCGCAATATGCCATCTTCAGTTAATTAAGGCTGATTACCT  240
NCIMB8817       AGACACTTGCCGTCAGGGATTTGCAATATGCCATCTTCAGTTAATTAAGGCTGATTACCT  240
DSM20492        AGACACTTGCCGTCAGGGATTCGCAATATGCCATCTTCAGTTAATTAAGGCTGATTACCT  240
CCUG47171       AGACACTTGCCGTCAGGGATTCGCAATATGCCATCTTCAGTTAATTAAGGCTGATTACCT  240
CCUG44481       AGACACTTACCGTCAGGGATTCGCAATATGCCATCTTCAGTTAATTAAGGCTGATTACCT  239
01M14315        AGACACTTGCCGTCAGGGATTCGCAATATACCATCTTTAGTTAATTAAGGCTGATTACCT  240
CCUG43299       AGACACTTGCCGTCAGGGATTCGCAATATACCATCTTTAGTTAATTAAGGCTGATTACCT  240
JCM1040         AGACACTTGCCGTCAGGGATTCGCAATATACCATCTTTAGTTAATTAAGGCTGATTACCT  240
DSM20555T       AGACACTTGCCGTCAGGGATTCGCAATATACCATCTTTAGTTAATTAAGGCTGATTACCT  240
Gul1            AGACACTTGCCGTCAGGGATTCGCAATATACCATCTTTAGTTAATTAAGGCTGATTACCT  240
Gul2            AGACACTTGCCGTCAGGGATTCGCAATATACCATCTTTAGTTAATTAAGGCTGATTACCT  240
JCM1047         AGACACTTGTCGTCAGGGATTCGCAATATACCATCTC-AGTTAATTAAGGCTGATTACCT  239
CCUG38008       AGACACTTGCCGTCAGGGATTCGCAATATGCCATCTTCAGTTAATTAAGGCTGATTACCT  227
LMG14476        AGACACTTACCGTCAGGGATTCGTAATATGCCATCTCCAGTTAATTAAGGCTGATTACCT  240
LMG14477        AGACACTTACCGTCAGGGATTCGTAATATGCCATCTCCAGTTAATTAAGGCTGATTACCT  240
                ********. *********** * *****.******  **********************
```

```
UCC118        TAGGAACCAAAAGAAGAATATCAACTAAGAATTCGTGTCGTGAAGTTGTGGTGACGCAAC  299
UCC119        TAGGAACCAAAAGAAGAATATCAACTAAGAATTCGTGTCGTGAAGTTGTGGTGACGCAAC  299
AH4231        TAGGAACCAAAAGAAGAATATCAACTAAGAATTCGTGTCGTGAAGTTGTGGTGACGCAAC  299
AH4331        TAGGAACCAAAAGAAGAATATCAACTAAGAATTCGTGTCGTGAAGTTGTGGTGACGCAAC  299
AH43310       TAGGAACCAAAAGAAGAATATCAACTAAGAATTCGTGTCGTGAAGTTGTGGTGACGCAAC  299
AH43324       TAGGAACCAAAAGAAGAATATCAACTAAGAATTCGTGTCGTGAAGTTGTGGTGACGCAAC  299
AH43348       TAGGAACCAAAAGAAGAATATCAACTAAGAATTCGTGTCGTGAAGTTGTGGTGACGCAAC  299
CCUG45735     TAGGAACCAAAAGAAGAATATCAACTAAGAATTCGTGTCGTGAAGTTGTGGTGACGCAAC  299
CCUG47825     TAGGAACCAAAAGAAGAATATCAACTAAGAATTCGTGTCGTGAAGTTGTGGTGACGCAAC  299
CCUG47826     TAGGAACCAAAAGAAGAATATCAACTAAGAATTCGTGTCGTGAAGTTGTGGTGACGCAAC  299
L21           TAGGAACCAAAAGAAGAATATCAACTAAGAATTCGTGTCGTGAAGTTGTGGTGACGCAAC  299
NCIMB8818     TAGGAACCAAAAGAAGAATATCAACTAAGAATTCGTGTCGTGAAGTTGTGGTGACGCAAC  299
JCM1046       TAGGAACCAA-AGAAGAATATCAATTAAGAATTCGTGTCGTGAAGTTGTGGTAACGCAAC  299
NCIMB8817     TAGGAACCAA-AGAAGAATATCAATTAAGAATTCGTGTCGTGAAGTTGTGGTAACGCAAC  299
DSM20492      TAGGAACCAA-AGAAGAATATCAATTAAGAATTCGTGTCGTGAAGTTGTGGTAACGCAAC  299
CCUG47171     TAGGAACCAAAAGAAGAAT-----------ATTCGTGTCGTGAAGTTGTGGTGACGCAAC  289
CCUG44481     TAGGAACCAA-AGAAGAATATCAATTAAGAATTCGTGTCGTGAAGTTGTGGTAACGTAAC  298
01M14315      TAGGAACCA-ACGAAGAATGTCAACTAAGAATTCGTGTCGTGAAGTTGTGGTGACACAGC  299
CCUG43299     TAGGAACCA-ACGAAGAATGTCAACTAAGAATTCGTGTCGTGAAGTTGTGGTGACACAGC  299
JCM1040       TAGGAACCA-ACGAAGAATGTCAACTAAGAATTCGTGTCGTGAAGTTGTGGTGACACAGC  299
DSM20555T     TAGGAACCAA-CGAAGAATATCAATTAAGAATTCGTGTCGTGAAGTTGTGGTGATGCAAC  299
Gul1          TAGGAACCAA-CGAAGAATATCAATTAAGAATTCGTGTCGTGAAGTTGTGGTGATGCAAC  299
Gul2          TAGGAACCA-ACGAAGAATATCAATTAAGAATTCGTGTCGTGAAGTTGTGGTGATGCAAC  299
JCM1047       TAGGAACCAA-AGAAGAATATCAACTAAGAATTCGTGTCGTGAAGTTGTGGTGACACAGC  298
CCUG38008     TAGGAACCAA-AGAAGAATATCAACTAAGAATTCGTGTCGTGAAGTTGTGGTGACACAGC  286
LMG14476      TAGGAACCAAAAGAAGAATATCAATTAAGAATTCGTGTCGTGAAGTTGTAGCGATGCAAC  300
LMG14477      TAGGAACCAAAAGAAGAATATCAATTAAGAATTCGTGTCGTGAAGTTGTAGCGATGCAAC  300
              *********  .*******           *******************.* .* . *.*


UCC118        TATAACTGACGAGTAAGGTTTGAGTAGCCAAAATCGACAATCTTACATTAACTG------  353
UCC119        TATAACTGACGAGTAAGGTTTGAGTAGCCAAAATCGACAATCTTACATTAACTG------  353
AH4231        TATAACTGACGAGTAAGGTTTGAGTAGCCAAAATCGACAATCTTACATTAACTG------  353
AH4331        TATAACTGACGAGTAAGGTTTGAGTAGCCAAAATCGACAATCTTACATTAACTG------  353
AH43310       TATAACTGACGAGTAAGGTTTGAGTAGCCAAAATCGACAATCTTACATTAACTG------  353
AH43324       TATAACTGACGAGTAAGGTTTGAGTAGCCAAAATCGACAATCTTACATTAACTG------  353
AH43348       TATAACTGACGAGTAAGGTTTGAGTAGCCAAAATCGACAATCTTACATTAACTG------  353
CCUG45735     TATAACTGACGAGTAAGGTTTGAGTAGCCAAAATCGACAATCTTACATTAACTG------  353
CCUG47825     TATAACTGACGAGTAAGGTTTGAGTAGCCAAAATCGACAATCTTACATTAACTG------  353
CCUG47826     TATAACTGACGAGTAAGGTTTGAGTAGCCAAAATCGACAATCTTACATTAACTG------  353
L21           TATAACTGACGAGTAAGGTTTGAGTAGCCAAAATCGACAATCTTACATTAACTG------  353
NCIMB8818     TATAACTGACGAGTAAGGTTTGAGTAGCCAAAATCGACAATCTTACATTAACTG------  353
JCM1046       TATAACTGACGAGTAAGGTTTGAGTAGCCAAAATCGACAATCTTACATTAACTG------  353
NCIMB8817     TATAACTGACGAGTAAGGTTTGAGTAGCCAAAATCGACAATCTTACATTAACTG------  353
DSM20492      TATAACTGACGAGTAAGGTTTGAGTAGCCAAAATCGACAATATTACATTAACTG------  353
CCUG47171     TATAACTGACGAGTAAGGTTTGAGTAGCCAAAATCGACAATCTTACATTAACTG------  343
CCUG44481     TATAACTGACGAGTAAGGTTTGAGTAGCCAAAATCGACAATATTACATTAATTG------  352
01M14315      TATAACTGACGAGTAAGGTTTGAGTAGCCAAAATCGACAATATTACATTAACTG------  353
CCUG43299     TATAACTGACGAGTAAGGTTTGAGTAGCCAAAATCGACAATATTACATTAACTG------  353
JCM1040       TATAACTGACGAGTAAGGTTTGAGTAGCCAAAATCGACAATATTACATTAACTG------  353
DSM20555T     TATAACTGACGAGTAAGGTTTGAGTAGCCAAAATCGACAATCTTATATTAACAGAAATAT  359
Gul1          TATAACTGACGAGTAAGGTTTGAGTAGCCAAAATCGACAATCTTATATTAACAGAAATAT  359
Gul2          TATAACTGACGAGTAAGGTTTGAGTAGCCAAAATCGACAATCTTATATTAACAGAAATAT  359
JCM1047       TATAACTGACGAGTAAGGTTTGAGTAGCCAAAATCGACAATATTACATTAACTG------  352
CCUG38008     TATAACTGACGAGTAAGGTTTGAGTAGCCAAAATCGACAATATTACATTAACTG------  340
LMG14476      TATAACTGACGAGTAAGGTTTGAGTAGCCAAAATCGACAATCTTACATTAAATT------  354
LMG14477      TATAACTGACGAGTAAGGTTTGAGTAGCCAAAATCGACAATCTTACATTAAATT------  354
              *****************************************.*** ***** :
```

```
UCC118        GAACATTGCACATGTTTGGTGAAAATTGGATAGGAAAAGATCTATGCCCTGAACTTGAAA  413
UCC119        GAACATTGCACATGTTTGGTGAAAATTGGATAGGAAAAGATCTATGCCCTGAACTTGAAA  413
AH4231        GAACATTGCACATGTTTGGTGAAAATTGGATAGGAAAAGATCTATGCCCTGAACTTGAAA  413
AH4331        GAACATTGCACATGTTTGGTGAAAATTGGATAGGAAAAGATCTATGCCCTGAACTTGAAA  413
AH43310       GAACATTGCACATGTTTGGTGAAAATTGGATAGGAAAAGATCTATGCCCTGAACTTGAAA  413
AH43324       GAACATTGCACATGTTTGGTGAAAATTGGATAGGAAAAGATCTATGCCCTGAACTTGAAA  413
AH43348       GAACATTGCACATGTTTGGTGAAAATTGGATAGGAAAAGATCTATGCCCTGAACTTGAAA  413
CCUG45735     GAACATTGCACATGTTTGGTGAAAATTGGATAGGAAAAGATCTATGCCCTGAACTTGAAA  413
CCUG47825     GAACATTGCACATGTTTGGTGAAAATTGGATAGGAAAAGATCTATGCCCTGAACTTGAAA  413
CCUG47826     GAACATTGCACATGTTTGGTGAAAATTGGATAGGAAAAGATCTATGCCCTGAACTTGAAA  413
L21           GAACATTGCACATGTTTGGTGAAAATTGGATAGGAAAAGATCTATGCCCTGAACTTGAAA  413
NCIMB8818     GAACATTGCACATGTTTGGTGAAAATTGGATAGGAAAAGATCTATGCCCTGAACTTGAAA  413
JCM1046       GAACATTGCACATGTTTAGTGAAAATTGGATAGGAAAAGGTCTATGCCCTGAACTTGAAA  413
NCIMB8817     GAACATTGCACATGTTTAGTGAAAATTGGATAGGAAAAGGTCTATGCCCTGAACTTGAAA  413
DSM20492      GAACATTGCACATGTTTGGTGAAAATTGGATAGGAAAAGGTCTATGCCCTGAACTTGAAA  413
CCUG47171     GAACATTGCACATGTTTGGTGAAAATTGGATAGGAAAAGGTCTATGCCCTGAACTTGAAA  403
CCUG44481     GAACATTGCACATGTTTGGTGAAAATTGGATAGGAAAAGGTCTATGCCCTGAACTTGAAA  412
01M14315      GAACATTGCACATGTTTGGTGAAAATTGGATAGGAAAAGGTCTATGCCCTGAACTTGAAA  413
CCUG43299     GAACATTGCACATGTTTGGTGAAAATTGGATAGGAAAAGGTCTATGCCCTGAACTTGAAA  413
JCM1040       GAACATTGCACATGTTTGGTGAAAATTGGATAGGAAAAGGTCTATGCCCTGAACTTGAAA  413
DSM20555T     GGACATTACACATGTTTGGTGAAAATTGGATAGGAAAAGGTCTATGCCCTGAACTTGAAA  419
Gul1          GGACATTACACATGTTTGGTGAAAATTGGATAGGAAAAGGTCTATGCCCTGAACTTGAAA  419
Gul2          GGACATTACACATGTTTGGTGAAAATTGGATAGGAAAAGGTCTATGCCCTGAACTTGAAA  419
JCM1047       GAACATTGCACATGTTTGGTGAAAATTGGATAGGAAAAGGTCTATGCCCTGAACTTGAAA  412
CCUG38008     GAACATTGCACATGTTTGGTGAAAATTGGATAGGAAAAGGTCTATGCCCTGAACTTGAAA  400
LMG14476      GGACATTACACATGTTTGGTGAAAGTTGGATAGGAAAAGGTCTATGCCCTGAACTCGAAA  414
LMG14477      GGACATTACACATGTTTGGTGAAAGTTGGATAGGAAAAGGTCTATGCCCTGAACTCGAAA  414
              *.*****.*********.******.**************.*************** ****
```

```
UCC118        TGCGGACTAGGTCGTGGAAGTTGCTAGCCATGATGTGCTAGTCTGATTCAGTAGTCTAAT  473
UCC119        TGCGGACTAGGTCGTGGAAGTTGCTAGCCATGATGTGCTAGTCTGATTCAGTAGTCTAAT  473
AH4231        TGCGGACTAGGTCGTGGAAGTTGCTAGCCATGATGTGCTAGTCTGATTCAGTAGTCTAAT  473
AH4331        TGCGGACTAGGTCGTGGAAGTTGCTAGCCATGATGTGCTAGTCTGATTCAGTAGTCTAAT  473
AH43310       TGCGGACTAGGTCGTGGAAGTTGCTAGCCATGATGTGCTAGTCTGATTCAGTAGTCTAAT  473
AH43324       TGCGGACTAGGTCGTGGAAGTTGCTAGCCATGATGTGCTAGTCTGATTCAGTAGTCTAAT  473
AH43348       TGCGGACTAGGTCGTGGAAGTTGCTAGCCATGATGTGCTAGTCTGATTCAGTAGTCTAAT  473
CCUG45735     TGCGGACTAGGTCGTGGAAGTTGCTAGCCATGATGTGCTAGTCTGATTCAGTAGTCTAAT  473
CCUG47825     TGCGGACTAGGTCGTGGAAGTTGCTAGCCATGATGTGCTAGTCTGATTCAGTAGTCTAAT  473
CCUG47826     TGCGGACTAGGTCGTGGAAGTTGCTAGCCATGATGTGCTAGTCTGATTCAGTAGTCTAAT  473
L21           TGCGGACTAGGTCGTGGAAGTTGCTAGCCATGATGTGCTAGTCTGATTCAGTAGTCTAAT  473
NCIMB8818     TGCGGACTAGGTCGTGGAAGTTGCTAGCCATGATGTGCTAGTCTGATTCAGTAGTCTAAT  473
JCM1046       TGCGGACTAGGTCGTGGAAGTTGCTAGCCATGATGTGCTAGTCTGATTCAGTAGTCTAAT  473
NCIMB8817     TGCGGACTAGGTCGTGGAAGTTGCTAGCCATGATGTGCTAGTCTGATTCAGTAGTCTAAT  473
DSM20492      TGCGGACTAGGTCATGGAAGTTGCTAGCCATGATGTGCTAGTCTGATTCAGTAGTCTAAT  473
CCUG47171     TGCGGACTAGGTCATGGAAGTTGCTAGCCATGATGTGCTAGTCTGATTCAGTAGTCTAAT  463
CCUG44481     TGCGGACTAGATCGTGGAAGTTGCTAGCCATGATGTGCTAGTCTGATTCAGTAGTCTAAT  472
01M14315      TGCGGACTAGGTCATGGAAGTTGCTAGCCATGATGTGCTAGTCTGATTCAGTAGTCTAAT  473
CCUG43299     TGCGGACTAGGTCATGGAAGTTGCTAGCCATGATGTGCTAGTCTGATTCAGTAGTCTAAT  473
JCM1040       TGCGGACTAGGTCATGGAAGTTGCTAGCCATGATGTGCTAGTCTGATTCAGTAGTCTAAT  473
DSM20555T     TGCGGACTAGGTCATGGAAGTTGCTAGCCATGATGTGCTAGTCTGATTCAGTAGTCTAAT  479
Gul1          TGCGGACTAGGTCATGGAAGTTGCTAGCCATGATGTGCTAGTCTGATTCAGTAGTCTAAT  479
Gul2          TGCGGACTAGGTCATGGAAGTTGCTAGCCATGATGTGCTAGTCTGATTCAGTAGTCTAAT  479
JCM1047       TGCGGACTAGGTCATGGAAGTTGCTAGCCATGATGTGCTAGTCTGATTCAGTAGTCTAAT  472
CCUG38008     TGCGGACTAGGTCATGGAAGTTGCTAGCCATGATGTGCTAGTCTGATTCAGTAGTCTAAT  460
LMG14476      TGCGGACTAGGTCATGGAAGTTGCTAGCCATGATGTGCTAGTTTGATTCAGTAGTCTAAT  474
LMG14477      TGCGGACTAGGTCATGGAAGTTGCTAGCCATGATGTGCTAGTTTGATTCAGTAGTCTAAT  474
              **********.**.**************************** *****************
```

```
UCC118          AAGTCCAGATACAAGAGTATCCCACGTAAGCCAATACGCGTCGGTTT    520
UCC119          AAGTCCAGATACAAGAGTATCCCACGTAAGCCAATACGCGTCGGTTT    520
AH4231          AAGTCCAGATACAAGAGTATCCCACGTAAGCCAATACGCGTCGGTTT    520
AH4331          AAGTCCAGATACAAGAGTATCCCACGTAAGCCAATACGCGTCGGTTT    520
AH43310         AAGTCCAGATACAAGAGTATCCCACGTAAGCCAATACGCGTCGGTTT    520
AH43324         AAGTCCAGATACAAGAGTATCCCACGTAAGCCAATACGCGTCGGTTT    520
AH43348         AAGTCCAGATACAAGAGTATCCCACGTAAGCCAATACGCGTCGGTTT    520
CCUG45735       AAGTCCAGATACAAGAGTATCCCACGTAAGCCAATACGCGTCGGTTT    520
CCUG47825       AAGTCCAGATACAAGAGTATCCCACGTAAGCCAATACGCGTCGGTTT    520
CCUG47826       AAGTCCAGATACAAGAGTATCCCACGTAAGCCAATACGCGTCGGTTT    520
L21             AAGTCCAGATACAAGAGTATCCCACGTAAGCCAATACGCGTCGGTTT    520
NCIMB8818       AAGTCCAGATACAAGAGTATCCCACGTAAGCCAATACGCGTCGGTTT    520
JCM1046         AAGTCCAGATACAAGAGTATCCCACGTAAGCCAATATGCGTCGGTTT    520
NCIMB8817       AAGTCCAGATACAAGAGTATCCCACGTAAGCCAATATGCGTCGGTTT    520
DSM20492        AAGTCCAGATACAAGAGTATCCCACGTAAACCAATACGCGTCGGTTT    520
CCUG47171       AAGTCCAGATACAAGAGTATCCCACGTAAGCCAATACGCGTCGGTTT    510
CCUG44481       AAGTCCAGATACAAGAGTATCCCACGTAAGCCAATACGCGTCGGTTT    519
01M14315        AAGTCCAGATACAAGAGTATCCCACGTAAGCCAATACGCGTCGGTTT    520
CCUG43299       AAGTCCAGATACAAGAGTATCCCACGTAAGCCAATACGCGTCGGTTT    520
JCM1040         AAGTCCAGATACAAGAGTATCCCACGTAAGCCAATACGCGTCGGTTT    520
DSM20555T       AAGTCCAGATACAAGAGTATCCCACGTAAGCCAATACGCGTCGGTTT    526
Gul1            AAGTCCAGATACAAGAGTATCCCACGTAAGCCAATACGCGTCGGTTT    526
Gul2            AAGTCCAGATACAAGAGTATCCCACGTAAGCCAATACGCGTCGGTTT    526
JCM1047         AAGTCCAGATACAAGAGTATCCCGCGTAAACCAATACGCGTCGGTTT    519
CCUG38008       AAGTCCAGATACAAGAGTATCCCACGTAAGCCAATACGCGTCGGTTT    507
LMG14476        AAGTCCAGATACAAGAGTATTCCACGTAAACCAATACGCGTCGGTTT    521
LMG14477        AAGTCCAGATACAAGAGTATTCCACGTAAACCAATACGCGTCGGTTT    521
                ******************** **.*****.****** **********
```

FIGURE S4: Multiple alignment of the lncRNA in *L. salivarius*.

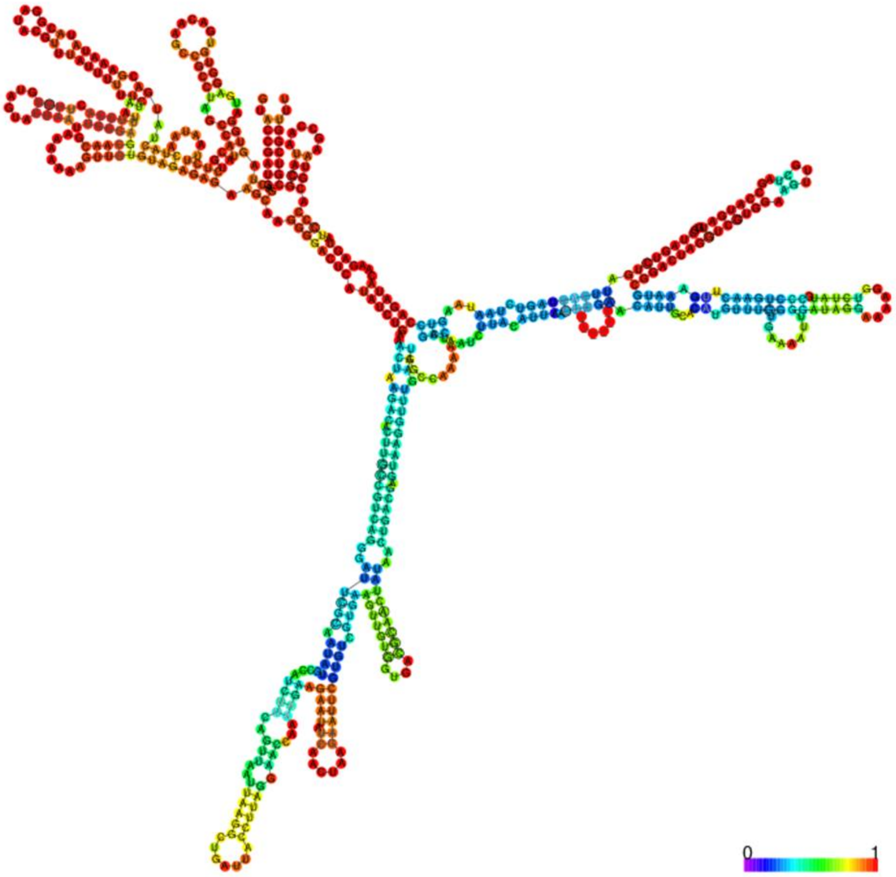

FIGURE S5: Structure drawing encoding base-pair probabilities of the lncRNA in *L. salivarius*. This structure was predicted with RNAalifold using the lncRNA alignment of the 27 *L. salivarius* strains. The free energy of the thermodynamic ensemble is -216.60 kcal/mol.

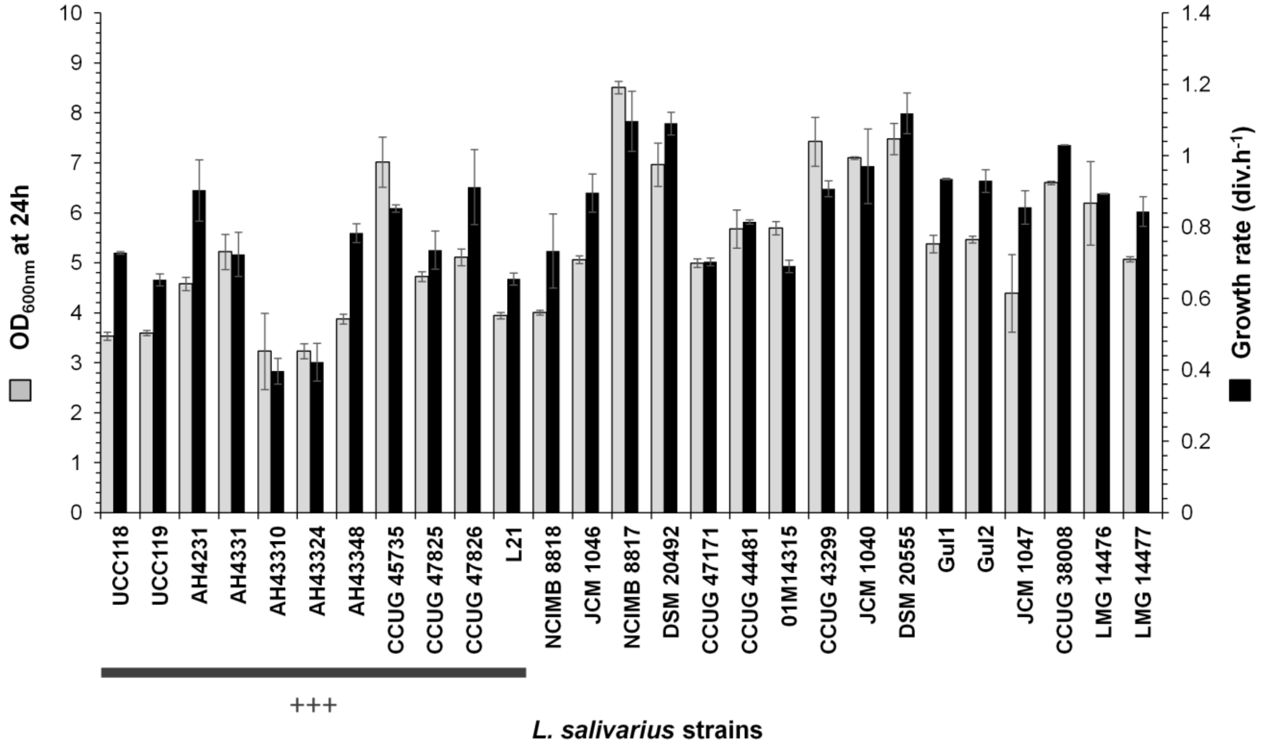

FIGURE S6: Growth of the 27 *L. salivarius* strains. Final OD_{600nm} (24h, grey) and growth rate (black) of the 27 *L. salivarius* strains used in this study were plotted. The 11 strains sharing a nucleotide sequence identical to that of *L. salivarius* UCC118 for the lncRNA region are marked with "+++". Data are means ± SD of 3 independent replicates.

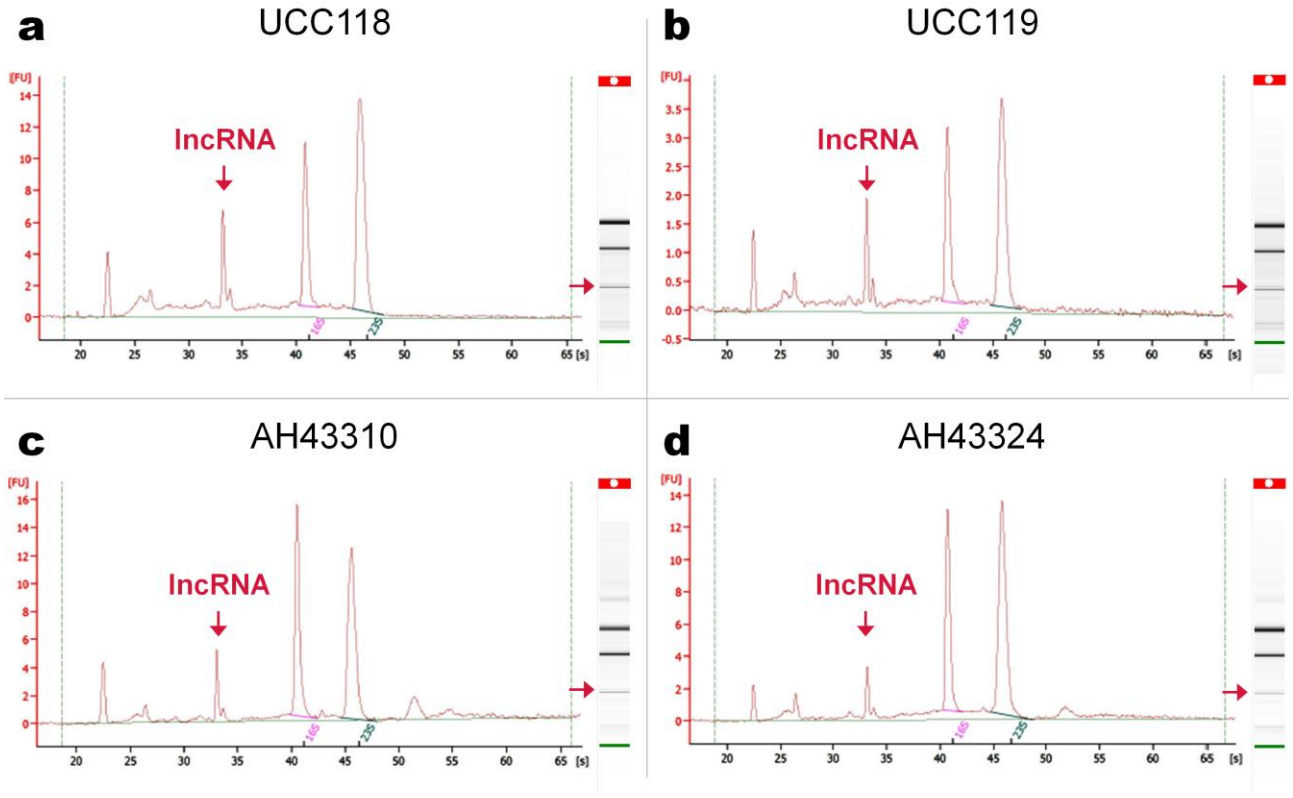

FIGURE S7: RNA profiles of the 4 *L. salivarius* strains with the highest expression levels of the lncRNA. RNA electropherograms of *L. salivarius* UCC118 **(A)**, UCC119 **(B)**, AH43310 **(C)** and AH43324 **(D)** in stationary phase (10h) obtained from the Agilent 2100 Bioanalyzer System. The lncRNA specific peak is highlighted in dark pink.

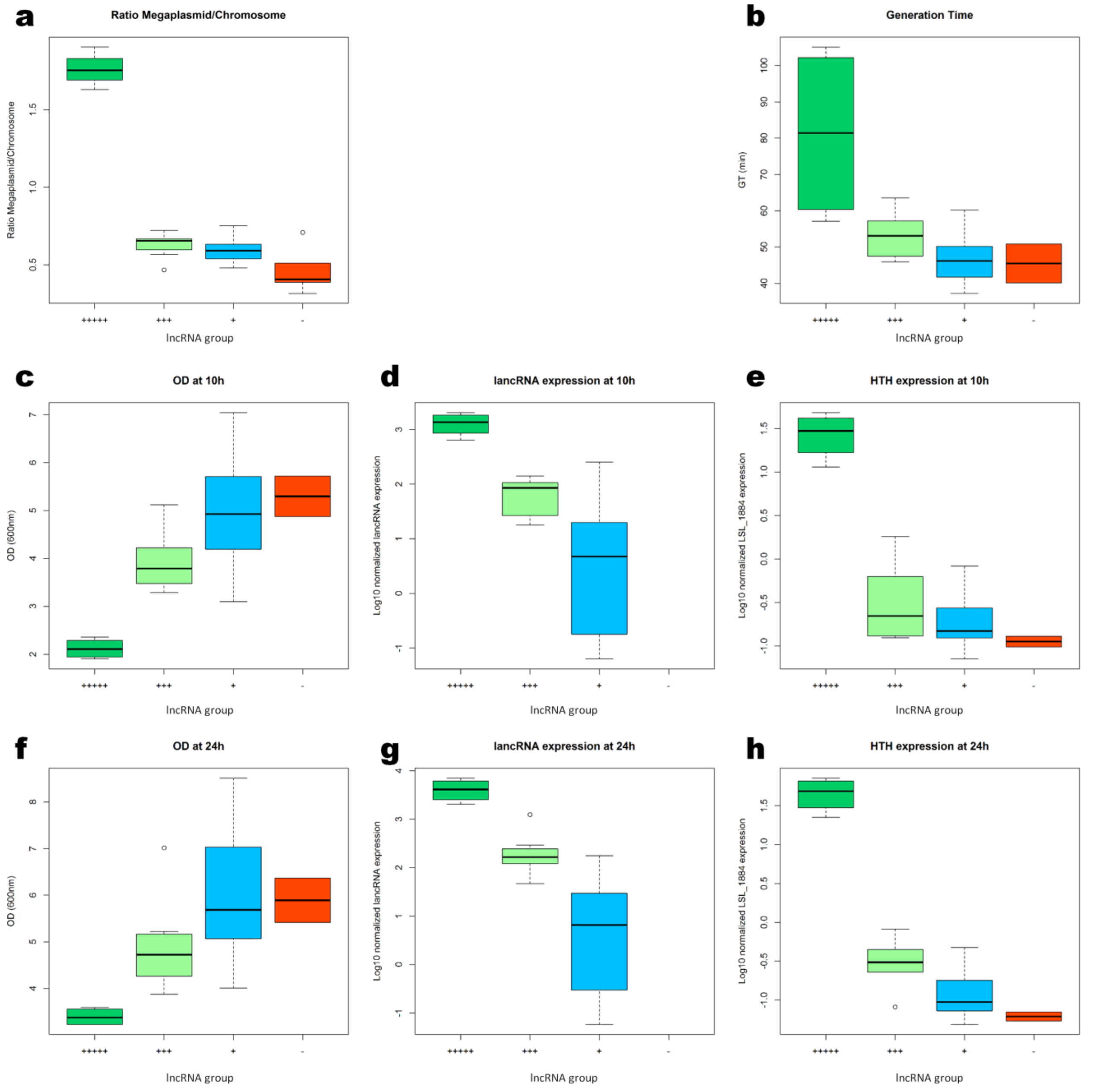

FIGURE S8: Comparison of data of 33 *L. salivarius* strains. The 33 *L. salivarius* strains were divided into 4 groups: +++++ for the strains with very high expression of the lncRNA, +++ for the strains with sequence 100% identical to the *L. salivarius* UCC118 lncRNA, + for the strains harboring a lncRNA sequence with SNPs/gaps and - for the strains without lncRNA sequence in their genome (Table 1). **(A)** Ratio megaplasmid/chromosome, **(B)** generation time, **(C)** OD (600nm) at 10h, **(D)** normalized expression of the lncRNA at 10h, **(E)** normalized expression of the HTH at 10h, **(F)** OD (600nm) at 24h, **(G)** normalized expression of the lncRNA at 24h, **(H)** normalized expression of the HTH at 24h.

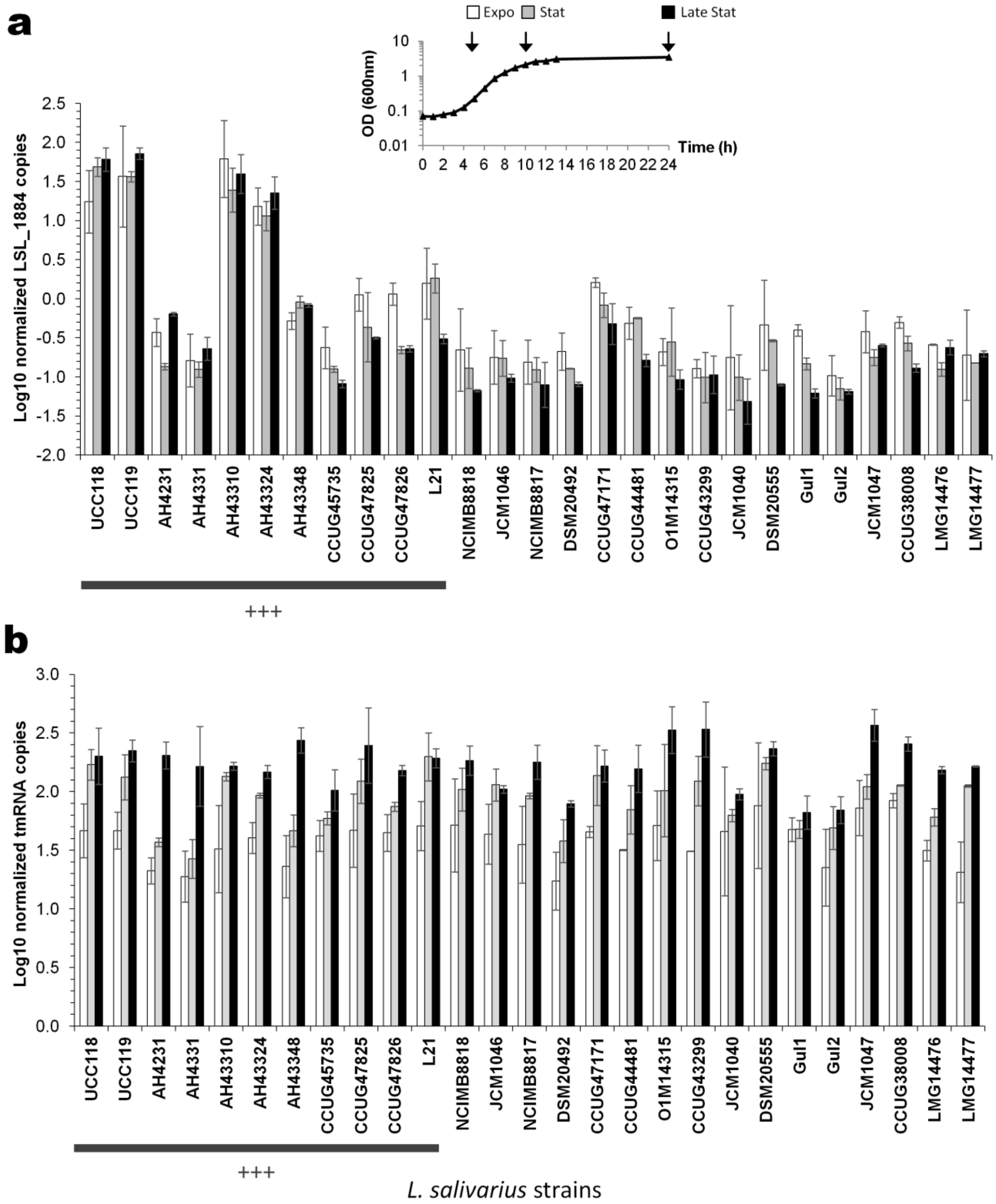

FIGURE S9: Expression levels of HTH and tmRNA during the growth of 27 *L. salivarius* strains. The expression levels of **(A)** HTH (*LSL_1884* in *L. salivarius* UCC118) and **(B)** tmRNA were quantified by RT-qPCR after 5 h (exponential phase, white), 10 h (stationary phase, grey) and 24 h (late stationary phase, black) of culture in MRS. The 11 strains sharing a nucleotide sequence identical to that of *L. salivarius* UCC118 for the lncRNA region are marked with “+++”. Data are means ± SD of 2 independent replicates.

Table S1: List of the primers used in this study for the quantitative PCR and the circular RACE

Name	Locus	Sequence targeted	Sequence (5'-3')	Size (bp)
L. salivarius genes				
lncRNA-qF<br>*lncRNA-qR*	LSL_1885	lncRNA	CTACTATGACGAAATATACGGATACG<br>GTGTCTTAGTTTCAGATAYGAGTCC	126
tail-qF<br>*tail-qR*	-	Tail of the lncRNA	AGGTTTGAGTAGCCAAAATCGAC<br>AGCACATCATGGCTAGCAAC	144
hth-qF<br>*hth-qR*	LSL_1884	Helix-turn-helix	CAATCCATTYATAARTGGTTATCTGG<br>GTATGTARAAYCTTACTGAGAATGAC	80
tmRNA-qF<br>*tmRNA-qR*	tmRNA	transfer-messenger RNA	ACTTTTGCGGCGTGAGAAAG<br>TCGCCACCTAAACCTCTACG	126
16SrDNA-qF<br>*16SrDNA-qR*	r16S	Small subunit of ribosome	TGGTAGTCCACGCCGTAAAC<br>CCAGGCGGAATGCTTATTGC	90
pflA-qF<br>*pflA-qR*	LSL_1872	pyruvate formate-lyase activating enzyme	CTTTTGGGGCGACAAAGGTG<br>ATGGCTGTCCACAAGTGTCC	134
pflD-qF<br>*pflD-qR*	LSL_1873	formate acetyltransferase	CTTCGGAGCTCGTGCAAATC<br>TGGTTCGTATGCAGGACCTAC	100
LSL0450-qF<br>*LSL0450-qR*	LSL_0450	MarR family transcriptional regulator	CGAAAAGGTTATGCTGAACGC<br>TGTGCTCGATAAACTAGTCTACC	95
fabD-qF<br>*fabD-qR*	LSL_0453	malonyl-CoA-[acyl-carrier-protein]-transacylase	GCTGTTGAAGGCATGGTTGG<br>GCATCATCTTGCATGTAAATAGCAC	125
era-qF<br>*era-qR*	LSL_0895	Ribosome-associated GTPase	TTTCCTATTTCTGCTTTGGAAGG<br>CAGCAGGATAGTACTGTGGAC	94
fusA-qF<br>*fusA-qR*	LSL_0202	Elongation factor	CGACGCTGGTAAGACAACAAC<br>GCACCATCGTGTGTTTCACC	84
groEL-qF<br>*groEL-qR*	LSL_1211	Chaperone	ACACGTGCTGCTGTTGAAGA<br>ACGTCGCCTTCTTCTTCCAA	98
gyrA-qF<br>*gyrA-qR*	LSL_0006	DNA gyrase subunit A	TGGGGCTACAGGTATTGCTG<br>GTCCAGGGATTGCCTCCATC	137
ileS-qF<br>*ileS-qR*	LSL_1042	Isoleucyl-tRNA synthetase	AAGCTGGTGTTGATCGTAAAGC<br>CACCAGCAACACCTAAACGC	120
recA-qF<br>*recA-qR*	LSL_1130	Recombinase A	GGATCCTGCATATGCTACTGC<br>GCGGCTACTGAGTCAACAAC	147
rpoB-qF<br>*rpoB-qR*	LSL_0197	RNA polymerase subunit β	ACGGGGCAGAACGTGTTATT<br>AACCAAGCCCCACGATTAGG	127
repA-qF<br>*repA-qR*	LSL_1739	Replication protein A	GCCAAAGGATTCGTAACTAACTTTG<br>GTGTTCTCTAGCTAAACGAAAAGC	93
pepN-qF<br>*penN-qR*	LSL_1745	Aminopeptidase N	ACTACATTTGCGCGTCAAGC<br>CGTCAAACTTTATAGCAAGATCGAAAG	82
ZntA-qF<br>*ZntA-qR*	LSL_1786	Lead, cadmium, zinc and mercury transporting ATPase	GTAGCCTTTGTTGGAGATGGC<br>TCAATCGCTACGTCCGTTCC	95
Circular RACE				
lncRNA_1F<br>*lncRNA_1R*		circularized lncRNA	GGAACATTGCACATGTTTGGTG<br>GCATATTGCGAATCCCTGACG	
lncRNA_2F<br>*lncRNA_2R*		circularized lncRNA	AGGTCGTGGAAGTTGCTAGC<br>TGAGTCCCCTTGCTTCTCTC	
Lactobacilli quantification				
F-Lacto 05<br>*R-Lacto 04*	r16S	Small subunit of ribosome	AGCAGTAGGGAATCTTCCA<br>CGCCACTGGTGTTCYTCCATATA	375

Table S2: Bacterial strains, plasmids and primers used for the mutant constructions of L. salivarius UCC118

**Bacterial strains or plasmid**	**Relevant characteristics**[a]	**Reference**
Escherichia coli		
EC101	$RepA^+$, Em^R, $pORI_{19}$ host strain	(1)
Lactococcus lactis		
LL108	$RepA^+$, Em^R, $pORI_{19}$ host strain	(2)
Lactobacillus salivarius		
UCC118 wild-type (WT)	Ileo-cecal isolate from a human adult	(3)
UCC118 pVE_{6007}	Strain harboring pVE_{6007} plasmid (Cm^R, $repA^{TS}$)	(4)
UCC118 *ΔlncRNA*	Clean deletion of the lncRNA	This study
UCC118 *ΔlncRNAΔLSL_1884*	Clean deletion of the lncRNA and the *LSL_1884* gene	This study
Plasmids		
$pORI_{19}$	Em^R, Ori^+, $RepA^-$, *lacZ'* deritive of $pORI_{28}$	(1)
$pORI_{19}$-*ΔlncRNA*	$pORI_{19}$ containing flanking regions of the lncRNA	This study
$pORI_{19}$-*ΔlncRNAΔLSL_1884*	$pORI_{19}$ containing flanking regions of the lncRNA and *LSL_1884* gene	This study
**Primer names**	**Sequence (5'-3')**[b]	**PCR amplicon**
WR_KO_US_F_EcoRI	*CGCGC***GAATTC**TGATCGTGTTTTAGGCGGTAC	Upstream fragments
WR_KO_US_F_BamHI	*ATC***GGATCC**TGATCGTGTTTTAGGCGGTAC	Upstream fragments
WR_KO1_US_R	CTCAATTTGAAGCTTCTAGTGTCTTCATCAATTATTCAACTC	ΔlncRNA upstream fragment
WR_KO1_DS_F	AGACACTAGAAGCTTCAAATTGAGGTGATTTCATGGAG	ΔlncRNA downstream fragment
WR_KO1_R_BamHI	*AGC***GGATCC**AGTCACAAATGATGGTTGATCAG	ΔlncRNA downstream fragment
WR_KO1_R_EcoRI	*CGCGC***GAATTC**AGTCACAAATGATGGTTGATCAG	ΔlncRNA downstream fragment
WR_KO2_US_R	ATTGTAGAGAAGCTTCTAGTGTCTTCATCAATTATTCAACTC	ΔlncRNA ΔHTH upstream fragment
WR_KO2_DS_F	AGACACTAGAAGCTTCTCTACAATAGTTCTAGCCTAGAAGC	ΔlncRNA ΔHTH downstream fragment
WR_KO2_DS_R_BamHI	*AGA***GGATCC**ACAACAGGTGTACTCATACCG	ΔlncRNA ΔHTH downstream fragment
WR_KO2_DS_R_EcoRI	*ATATC***GAATTC**ACAACAGGTGTACTCATACCG	ΔlncRNA ΔHTH downstream fragment
LSL_1886_F	CTGCTTCCGGTGGTTTTGG	Clean deletion verification
LSL_1883_R	TGCTGTTCCTTTAGCTTTAAATTCG	Clean deletion verification

[a] Em^R, erythromycin resistant; Ori^+, replication origin; $RepA^-$, lacking replication protein A; Cm^R, chloramphenicol resistant; ^{TS}, temperature sensitive;
[b] Sequences in bold, EcoRI or BamHI restriction sites used for plasmid constructions; italics, clamp; highlighted, overlaps allowing the SOE-PCR of upstream and downstream fragments; underlined, HindIII restriction site allowing the quick verification of the successful SOE amplifications.

REFERENCES

1. **Law J, Buist G, Haandrikman A, Kok J, Venema G, Leenhouts K**. 1995. A system to generate chromosomal mutations in *Lactococcus lactis* which allows fast analysis of targeted genes. J Bacteriol **177**:7011-8.
2. **Leenhouts K, Bolhuis A, Venema G, Kok J**. 1998. Construction of a food-grade multiple-copy integration system for *Lactococcus lactis*. Appl Microbiol Biotechnol **49**:417-23.
3. **Dunne C, Murphy L, Flynn S, O'Mahony L, O'Halloran S, Feeney M, Morrissey D, Thornton G, Fitzgerald G, Daly C, Kiely B, Quigley EMM, O'Sullivan GC, Shanahan F, Collins JK**. 1999. Probiotics: from myth to reality. Demonstration of functionality in animal models of disease and in human clinical trials. Antonie Van Leeuwenhoek **76**:279-292.
4. **van Pijkeren JP, Canchaya C, Ryan KA, Li Y, Claesson MJ, Sheil B, Steidler L, O'Mahony L, Fitzgerald GF, van Sinderen D, O'Toole PW**. 2006. Comparative and functional analysis of sortase-dependent proteins in the predicted secretome of *Lactobacillus salivarius* UCC118. Appl Environ Microbiol **72**:4143-4153.

Table S3: Relative gene expression levels for the 27 L. salivarius strains

Strain	Growth phase	*LSL_1885*	*lncRNA tail*	*LSL_1884*	*tmRNA*	*16S*	*pflA*	*pflD*	*LSL0450*	*fabD*
UCC118	Expo	2.65 ± 0.26	2.44 ± 0.15	1.24 ± 0.40	1.67 ± 0.23	3.23 ± 0.24	0.02 ± 0.19	0.42 ± 0.05	0.09 ± 0.13	0.16 ± 0.03
	Stat	3.39 ± 0.07	3.23 ± 0.30	1.69 ± 0.12	2.23 ± 0.13	3.16 ± 0.11	0.48 ± 0.12	0.69 ± 0.12	0.07 ± 0.39	0.16 ± 0.20
	LateStat	3.66 ± 0.03	3.80 ± 0.53	1.78 ± 0.15	2.30 ± 0.24	3.63 ± 0.53	1.78 ± 0.21	1.97 ± 0.15	-0.40 ± 0.07	-0.16 ± 0.24
UCC119	Expo	2.96 ± 0.51	2.82 ± 0.38	1.57 ± 0.65	1.67 ± 0.16	3.53 ± 0.46	-0.07 ± 0.28	0.26 ± 0.23	-0.32 ± 0.26	-0.03 ± 0.11
	Stat	3.29 ± 0.02	3.13 ± 0.00	1.56 ± 0.07	2.12 ± 0.19	3.12 ± 0.02	0.31 ± 0.19	0.57 ± 0.04	0.15 ± 0.06	0.16 ± 0.02
	LateStat	3.79 ± 0.05	3.91 ± 0.16	1.86 ± 0.08	2.35 ± 0.09	3.54 ± 0.13	1.89 ± 0.15	2.08 ± 0.15	-0.30 ± 0.04	-0.13 ± 0.03
AH4231	Expo	0.66 ± 0.19	0.42 ± 0.11	-0.43 ± 0.18	1.32 ± 0.11	3.03 ± 0.00	-1.47 ± 0.07	-1.15 ± 0.09	0.60 ± 0.04	0.67 ± 0.07
	Stat	1.76 ± 0.13	1.39 ± 0.25	-0.87 ± 0.04	1.57 ± 0.04	3.33 ± 0.11	0.66 ± 0.08	1.07 ± 0.21	-0.35 ± 0.34	-0.27 ± 0.37
	LateStat	2.54 ± 0.00	2.08 ± 0.30	-0.20 ± 0.02	2.31 ± 0.11	4.05 ± 0.17	1.10 ± 0.04	1.07 ± 0.11	-0.05 ± 0.26	0.22 ± 0.34
AH4331	Expo	0.39 ± 0.11	0.26 ± 0.73	-0.79 ± 0.34	1.28 ± 0.22	3.34 ± 0.44	-1.70 ± 0.57	-1.64 ± 0.17	0.86 ± 0.36	0.88 ± 0.20
	Stat	1.36 ± 0.09	1.14 ± 0.12	-0.90 ± 0.10	1.43 ± 0.16	3.02 ± 0.13	-0.30 ± 0.21	-0.02 ± 0.24	0.10 ± 0.09	0.17 ± 0.13
	LateStat	1.83 ± 0.03	2.30 ± 0.89	-0.64 ± 0.15	2.22 ± 0.34	3.97 ± 0.44	0.44 ± 0.11	0.64 ± 0.18	-0.05 ± 0.04	0.24 ± 0.09
AH43310	Expo	3.13 ± 0.54	2.86 ± 0.80	1.79 ± 0.49	1.51 ± 0.37	3.37 ± 0.50	-0.49 ± 0.14	-0.49 ± 0.15	-0.19 ± 0.31	0.14 ± 0.03
	Stat	3.10 ± 0.11	3.01 ± 0.30	1.39 ± 0.28	2.13 ± 0.04	3.26 ± 0.06	0.55 ± 0.17	0.84 ± 0.24	0.26 ± 0.06	0.33 ± 0.01
	LateStat	3.49 ± 0.26	3.50 ± 0.21	1.59 ± 0.25	2.22 ± 0.03	3.62 ± 0.28	1.52 ± 0.32	1.69 ± 0.33	-0.81 ± 0.30	-0.57 ± 0.40
AH43324	Expo	2.66 ± 0.15	2.28 ± 0.02	1.18 ± 0.24	1.61 ± 0.13	3.14 ± 0.03	-0.96 ± 0.18	-0.56 ± 0.13	0.04 ± 0.02	0.11 ± 0.13
	Stat	2.78 ± 0.10	2.83 ± 0.29	1.06 ± 0.19	1.97 ± 0.02	3.17 ± 0.25	0.36 ± 0.25	0.57 ± 0.24	0.09 ± 0.07	0.13 ± 0.04
	LateStat	3.28 ± 0.03	3.35 ± 0.41	1.35 ± 0.21	2.17 ± 0.06	3.56 ± 0.19	1.58 ± 0.02	1.72 ± 0.06	-0.80 ± 0.01	-0.56 ± 0.14
AH43348	Expo	1.14 ± 0.13	0.88 ± 0.24	-0.29 ± 0.11	1.36 ± 0.26	3.10 ± 0.02	-1.39 ± 0.24	-0.71 ± 0.24	0.46 ± 0.31	0.45 ± 0.13
	Stat	2.15 ± 0.02	2.06 ± 0.12	-0.04 ± 0.08	1.67 ± 0.13	3.24 ± 0.01	0.98 ± 0.00	1.22 ± 0.02	0.36 ± 0.08	0.35 ± 0.15
	LateStat	2.74 ± 0.04	3.44 ± 0.13	-0.08 ± 0.03	2.44 ± 0.11	4.31 ± 0.11	1.11 ± 0.08	1.00 ± 0.17	-0.42 ± 0.13	-0.14 ± 0.02
CCUG45735	Expo	0.16 ± 0.32	0.35 ± 0.08	-0.63 ± 0.26	1.62 ± 0.13	3.24 ± 0.42	-1.85 ± 0.16	-1.70 ± 0.08	0.67 ± 0.25	0.82 ± 0.37
	Stat	1.34 ± 0.20	1.21 ± 0.20	-0.90 ± 0.04	1.77 ± 0.06	3.34 ± 0.15	-0.04 ± 0.23	0.27 ± 0.36	-0.39 ± 0.46	-0.28 ± 0.40
	LateStat	1.80 ± 0.07	1.54 ± 0.23	-1.09 ± 0.05	2.01 ± 0.18	3.80 ± 0.16	0.48 ± 0.06	0.38 ± 0.21	-0.72 ± 0.08	-0.38 ± 0.24
CCUG47825	Expo	1.31 ± 0.14	1.20 ± 0.17	0.05 ± 0.21	1.67 ± 0.31	2.90 ± 0.11	-1.70 ± 0.15	-1.56 ± 0.24	0.27 ± 0.14	0.35 ± 0.25
	Stat	2.09 ± 0.11	2.21 ± 0.46	-0.36 ± 0.44	2.09 ± 0.19	3.26 ± 0.06	0.33 ± 0.48	0.57 ± 0.43	-0.34 ± 0.07	-0.26 ± 0.07
	LateStat	2.55 ± 0.02	2.39 ± 0.01	-0.50 ± 0.01	2.39 ± 0.32	3.53 ± 0.11	0.91 ± 0.08	0.62 ± 0.29	-1.14 ± 0.09	-0.90 ± 0.18
CCUG47826	Expo	1.12 ± 0.03	1.31 ± 0.43	0.06 ± 0.14	1.65 ± 0.16	3.10 ± 0.26	-1.70 ± 0.10	-1.61 ± 0.05	0.49 ± 0.27	0.54 ± 0.17
	Stat	2.00 ± 0.11	1.91 ± 0.12	-0.66 ± 0.05	1.87 ± 0.04	3.23 ± 0.10	0.69 ± 0.16	0.93 ± 0.15	-0.69 ± 0.33	-0.57 ± 0.26
	LateStat	2.19 ± 0.02	2.24 ± 0.04	-0.64 ± 0.04	2.18 ± 0.04	3.68 ± 0.01	1.13 ± 0.07	1.18 ± 0.10	-1.08 ± 0.04	-0.88 ± 0.13
L21	Expo	1.18 ± 0.21	1.31 ± 0.52	0.19 ± 0.45	1.71 ± 0.21	3.12 ± 0.24	-1.65 ± 0.09	-1.46 ± 0.01	0.46 ± 0.02	0.58 ± 0.13
	Stat	1.95 ± 0.04	1.92 ± 0.08	0.26 ± 0.18	2.30 ± 0.20	3.36 ± 0.31	-0.10 ± 0.10	0.01 ± 0.14	0.23 ± 0.05	0.26 ± 0.13
	LateStat	2.06 ± 0.01	2.14 ± 0.17	-0.51 ± 0.06	2.29 ± 0.08	3.75 ± 0.11	0.85 ± 0.12	0.79 ± 0.02	-0.49 ± 0.10	-0.18 ± 0.17

NCIMB8818	Expo	-0.50 ± 0.24	-0.13 ± 0.06	-0.66 ± 0.53	1.71 ± 0.40	3.21 ± 0.34	-1.83 ± 0.15	-1.72 ± 0.23	-0.62 ± 0.21	-0.43 ± 0.00
	Stat	0.19 ± 0.02	-0.11 ± 0.14	-0.89 ± 0.26	2.02 ± 0.18	3.14 ± 0.17	0.27 ± 0.55	0.57 ± 0.43	-0.77 ± 0.25	-0.66 ± 0.21
	LateStat	0.31 ± 0.02	0.39 ± 0.39	-1.18 ± 0.01	2.27 ± 0.13	3.23 ± 0.01	1.11 ± 0.04	1.13 ± 0.08	-0.80 ± 0.09	-0.54 ± 0.12
JCM1046	Expo	0.00 ± 0.29	0.29 ± 0.43	-0.75 ± 0.34	1.64 ± 0.26	3.23 ± 0.16	-1.01 ± 0.19	-0.83 ± 0.28	0.53 ± 0.05	0.66 ± 0.18
	Stat	0.82 ± 0.15	0.75 ± 0.13	-0.76 ± 0.23	2.06 ± 0.14	3.47 ± 0.09	0.44 ± 0.00	0.67 ± 0.10	-0.17 ± 0.04	-0.02 ± 0.06
	LateStat	0.78 ± 0.08	0.81 ± 0.26	-1.02 ± 0.05	2.02 ± 0.03	3.73 ± 0.17	0.58 ± 0.08	0.63 ± 0.16	-0.39 ± 0.03	-0.11 ± 0.19
NCIMB8817	Expo	-0.10 ± 0.06	-0.03 ± 0.15	-0.81 ± 0.28	1.55 ± 0.33	3.08 ± 0.10	-0.99 ± 0.14	-0.75 ± 0.14	0.48 ± 0.01	0.59 ± 0.11
	Stat	-0.84 ± 0.12	-0.85 ± 0.27	-0.91 ± 0.16	1.96 ± 0.02	3.66 ± 0.20	0.72 ± 0.21	1.00 ± 0.11	-0.73 ± 0.45	-0.48 ± 0.34
	LateStat	-0.89 ± 0.04	-0.65 ± 0.48	-1.10 ± 0.29	2.25 ± 0.14	4.11 ± 0.26	0.99 ± 0.14	1.01 ± 0.16	-0.76 ± 0.21	-0.47 ± 0.12
DSM20492	Expo	0.63 ± 0.17	0.46 ± 0.17	-0.68 ± 0.24	1.24 ± 0.25	2.96 ± 0.21	-0.86 ± 0.25	-0.63 ± 0.22	0.46 ± 0.06	0.50 ± 0.01
	Stat	1.43 ± 0.09	1.43 ± 0.44	-0.89 ± 0.01	1.58 ± 0.18	3.31 ± 0.20	0.36 ± 0.06	0.55 ± 0.13	-0.28 ± 0.01	-0.14 ± 0.14
	LateStat	1.77 ± 0.06	1.61 ± 0.10	-1.09 ± 0.03	1.90 ± 0.03	3.57 ± 0.27	0.59 ± 0.13	0.61 ± 0.11	-0.02 ± 0.21	-0.57 ± 0.72
CCUG47171	Expo	1.17 ± 0.17	1.13 ± 0.05	0.21 ± 0.06	1.66 ± 0.05	3.07 ± 0.19	-1.71 ± 0.08	-1.59 ± 0.06	0.58 ± 0.04	0.70 ± 0.00
	Stat	2.16 ± 0.07	2.66 ± 0.58	-0.08 ± 0.16	2.14 ± 0.25	3.49 ± 0.32	-0.06 ± 0.30	0.28 ± 0.40	-0.18 ± 0.01	-0.07 ± 0.05
	LateStat	2.20 ± 0.14	2.28 ± 0.07	-0.32 ± 0.26	2.22 ± 0.14	3.41 ± 0.10	0.91 ± 0.11	1.06 ± 0.04	-0.52 ± 0.00	-0.29 ± 0.13
CCUG44481	Expo	0.06 ± 0.19	0.07 ± 0.45	-0.31 ± 0.21	1.50 ± 0.01	3.10 ± 0.09	-1.46 ± 0.08	-1.30 ± 0.01	0.82 ± 0.11	0.90 ± 0.08
	Stat	0.45 ± 0.12	0.69 ± 0.63	-0.25 ± 0.01	1.85 ± 0.21	3.39 ± 0.27	0.33 ± 0.17	0.70 ± 0.09	0.08 ± 0.06	0.22 ± 0.01
	LateStat	0.82 ± 0.09	0.85 ± 0.20	-0.79 ± 0.08	2.20 ± 0.20	3.81 ± 0.03	1.52 ± 0.19	1.74 ± 0.12	-0.41 ± 0.06	-0.24 ± 0.11
O1M14315	Expo	-1.58 ± 0.17	-1.11 ± 0.24	-0.68 ± 0.17	1.71 ± 0.30	3.13 ± 0.06	-1.89 ± 0.03	-1.81 ± 0.03	0.74 ± 0.02	0.87 ± 0.07
	Stat	-1.24 ± 0.06	-1.17 ± 0.16	-0.55 ± 0.44	2.01 ± 0.39	3.16 ± 0.09	0.29 ± 0.02	0.59 ± 0.17	0.31 ± 0.42	0.30 ± 0.26
	LateStat	-0.39 ± 0.16	-0.41 ± 0.06	-1.03 ± 0.12	2.52 ± 0.20	3.90 ± 0.05	1.31 ± 0.00	1.07 ± 0.05	-0.38 ± 0.03	-0.11 ± 0.02
CCUG43299	Expo	-1.74 ± 0.19	-1.86 ± 0.39	-0.89 ± 0.12	1.49 ± 0.00	2.96 ± 0.04	-1.76 ± 0.04	-1.46 ± 0.02	0.70 ± 0.26	0.78 ± 0.21
	Stat	-1.21 ± 0.02	-1.07 ± 0.42	-1.01 ± 0.32	2.09 ± 0.21	3.49 ± 0.15	0.80 ± 0.05	1.04 ± 0.17	-0.43 ± 0.33	-0.16 ± 0.29
	LateStat	-0.90 ± 0.07	-0.82 ± 0.14	-0.97 ± 0.24	2.53 ± 0.23	4.06 ± 0.15	-0.06 ± 0.12	0.34 ± 0.03	-0.08 ± 0.02	0.26 ± 0.04
JCM1040	Expo	-1.90 ± 0.11	-1.39 ± 0.75	-0.75 ± 0.67	1.66 ± 0.55	3.34 ± 0.51	-1.02 ± 0.58	-0.78 ± 0.58	0.47 ± 0.36	0.60 ± 0.34
	Stat	-1.27 ± 0.05	-1.04 ± 0.61	-1.01 ± 0.29	1.80 ± 0.05	3.63 ± 0.20	1.58 ± 0.04	1.83 ± 0.02	-0.88 ± 0.09	-0.70 ± 0.09
	LateStat	-1.26 ± 0.06	-1.22 ± 0.25	-1.31 ± 0.29	1.98 ± 0.05	3.69 ± 0.19	1.57 ± 0.09	1.38 ± 0.10	-0.78 ± 0.03	-0.46 ± 0.04
DSM20555	Expo	-0.07 ± 0.24	0.22 ± 0.48	-0.34 ± 0.58	1.88 ± 0.54	3.19 ± 0.04	-0.67 ± 0.08	-0.40 ± 0.08	0.39 ± 0.08	0.51 ± 0.08
	Stat	1.13 ± 0.23	1.13 ± 0.09	-0.54 ± 0.01	2.24 ± 0.05	3.79 ± 0.03	0.78 ± 0.00	1.00 ± 0.00	-0.25 ± 0.04	-0.07 ± 0.01
	LateStat	1.20 ± 0.12	1.45 ± 0.13	-1.10 ± 0.01	2.37 ± 0.06	4.36 ± 0.13	0.80 ± 0.03	0.73 ± 0.08	-0.33 ± 0.10	-0.10 ± 0.00
Gul1	Expo	0.31 ± 0.48	0.26 ± 0.32	-0.40 ± 0.07	1.68 ± 0.10	2.99 ± 0.01	-0.55 ± 0.03	-0.24 ± 0.06	0.41 ± 0.51	0.45 ± 0.60
	Stat	1.22 ± 0.01	1.34 ± 0.21	-0.83 ± 0.08	1.68 ± 0.08	3.04 ± 0.05	0.48 ± 0.02	0.72 ± 0.06	-1.00 ± 0.03	-0.89 ± 0.13
	LateStat	1.36 ± 0.02	1.43 ± 0.10	-1.21 ± 0.06	1.82 ± 0.14	3.46 ± 0.01	0.90 ± 0.13	0.83 ± 0.07	-1.01 ± 0.18	-0.82 ± 0.03
Gul2	Expo	-0.11 ± 0.14	-0.22 ± 0.10	-0.99 ± 0.26	1.35 ± 0.33	2.95 ± 0.02	-0.89 ± 0.03	-0.51 ± 0.02	0.75 ± 0.05	0.81 ± 0.19
	Stat	1.28 ± 0.12	1.34 ± 0.29	-1.15 ± 0.14	1.69 ± 0.18	3.12 ± 0.03	0.57 ± 0.19	0.82 ± 0.12	-1.26 ± 0.25	-1.14 ± 0.12

	LateStat	1.47 ± 0.05	1.46 ± 0.24	-1.19 ± 0.03	1.84 ± 0.12	3.38 ± 0.28	0.76 ± 0.04	0.75 ± 0.01	-1.10 ± 0.01	-0.86 ± 0.06
JCM1047	Expo	0.09 ± 0.22	0.46 ± 0.38	-0.42 ± 0.27	1.86 ± 0.24	3.24 ± 0.13	0.09 ± 0.31	0.32 ± 0.31	0.37 ± 0.08	0.54 ± 0.22
	Stat	-0.12 ± 0.12	-0.22 ± 0.04	-0.75 ± 0.10	2.04 ± 0.10	3.65 ± 0.02	1.49 ± 0.00	1.74 ± 0.02	-0.33 ± 0.15	-0.19 ± 0.01
	LateStat	-0.13 ± 0.37	0.31 ± 0.32	-0.60 ± 0.02	2.57 ± 0.14	4.45 ± 0.25	1.46 ± 0.07	1.28 ± 0.02	0.00 ± 0.19	0.22 ± 0.03
CCUG38008	Expo	-0.11 ± 0.09	-0.01 ± 0.31	-0.30 ± 0.07	1.92 ± 0.06	2.92 ± 0.24	-1.16 ± 0.18	-0.96 ± 0.23	0.55 ± 0.01	0.63 ± 0.01
	Stat	-0.76 ± 0.29	-0.54 ± 0.02	-0.57 ± 0.09	2.05 ± 0.01	3.40 ± 0.41	0.92 ± 0.27	1.09 ± 0.41	-0.33 ± 0.12	-0.20 ± 0.03
	LateStat	-0.91 ± 0.22	-0.38 ± 0.65	-0.89 ± 0.05	2.41 ± 0.06	4.26 ± 0.39	1.50 ± 0.02	1.47 ± 0.12	-0.93 ± 0.17	-0.59 ± 0.23
LMG14476	Expo	0.05 ± 0.05	0.47 ± 0.11	-0.59 ± 0.01	1.50 ± 0.09	3.30 ± 0.09	-0.98 ± 0.01	-0.80 ± 0.02	0.57 ± 0.04	0.73 ± 0.01
	Stat	1.34 ± 0.16	0.87 ± 0.05	-0.90 ± 0.09	1.78 ± 0.07	3.48 ± 0.02	1.24 ± 0.01	1.42 ± 0.02	-0.39 ± 0.04	-0.28 ± 0.16
	LateStat	1.76 ± 0.18	1.22 ± 0.01	-0.62 ± 0.09	2.18 ± 0.03	3.66 ± 0.04	1.21 ± 0.01	1.22 ± 0.07	-0.17 ± 0.02	-0.01 ± 0.02
LMG14477	Expo	0.32 ± 0.39	0.09 ± 0.44	-0.72 ± 0.58	1.31 ± 0.26	2.92 ± 0.04	-0.34 ± 0.01	-0.08 ± 0.06	0.58 ± 0.03	0.61 ± 0.01
	Stat	1.41 ± 0.04	1.36 ± 0.17	-0.82 ± 0.00	2.05 ± 0.01	3.90 ± 0.21	1.18 ± 0.04	1.41 ± 0.02	-0.18 ± 0.20	-0.02 ± 0.15
	LateStat	1.68 ± 0.09	1.27 ± 0.05	-0.70 ± 0.04	2.22 ± 0.01	3.90 ± 0.01	1.14 ± 0.02	1.28 ± 0.04	0.04 ± 0.08	0.21 ± 0.04

[a]*: All expression values are normalized and expressed in log10. The 11 strains highlighted in grey share 100% identity at the nucleotide level for the lncRNA.*

Table S4: Differences of gene expression between L. salivarius UCC118 wild-type and the mutants

Locus	Name	Predicted function	Genomic Position	ΔlncRNA vs WT		ΔlncRNA ΔHTH vs WT	
				Expo	Stat	Expo	Stat
Upregulated genes							
LSL_0152.1	#N/A	#N/A	Chromosome	2.47	2.60	3.07	1.76
LSL_0449	*fabA*	(3R)-hydroxyacyl-ACP dehydratase	Chromosome	3.88	1.43	2.69	1.42
LSL_0450	-	MarR family transcriptional regulator	Chromosome	3.80	1.63	2.63	1.51
LSL_0451	*fabH*	3-oxoacyl-ACP synthase	Chromosome	3.30	1.57	2.24	1.54
LSL_0452	*acpP*	acyl carrier protein	Chromosome	4.45	1.74	3.72	1.73
LSL_0453	*fabD*	malonyl-CoA-[acyl-carrier-protein]-transacylase	Chromosome	3.93	2.17	3.57	1.63
LSL_0454	*fabG*	3-ketoacyl-ACP reductase	Chromosome	3.21	1.58	2.73	1.61
LSL_0455	*fabB*	3-oxoacyl-ACP synthase	Chromosome	3.70	1.60	2.90	1.76
LSL_0456	*accB*	biotin carboxyl carrier protein of acetyl-CoA carboxylase	Chromosome	4.34	1.50	4.23	1.89
LSL_0457	*fabZ*	(3R)-hydroxymyristoyl-ACP dehydratase	Chromosome	2.73	1.42	2.25	1.48
LSL_0458	*accC*	biotin carboxylase	Chromosome	3.48	1.38	2.80	1.68
LSL_0459	*accD*	acetyl-coenzyme A carboxylase carboxyl transferase subunit beta	Chromosome	3.67	1.52	3.69	1.59
LSL_0460	*accA*	acetyl-coenzyme A carboxylase carboxyl transferase subunit alpha	Chromosome	2.70	1.28	2.59	1.33
LSL_0461	*fabI*	enoyl-ACP reductase	Chromosome	2.67	1.35	2.15	1.39
LSL_1136	-	23S rRNA methyltransferase	Chromosome	2.69	2.25	3.07	1.43
LSL_1831	-	hypothetical protein LSL_1831	pMP118	4.83	2.55	2.81	1.72
LSL_1832	-	hypothetical protein LSL_1832	pMP118	3.26	2.27	2.57	1.99
LSL_1846	-	hypothetical protein LSL_1846	pMP118	3.17	1.45	2.23	1.08
Downregulated genes							
LSL_0139	-	hypothetical protein LSL_0139	Chromosome	-1.10	-2.73	1.13	-3.08
LSL_0376	*lacZ*	beta-galactosidase	Chromosome	-3.32	-3.73	-1.44	-4.93
LSL_0864	-	hypothetical protein LSL_0864	Chromosome	-3.72	-3.00	-4.39	-2.57
LSL_1336	*wecD*	acetyltransferase	Chromosome	-5.50	-5.10	-65.30	-103.17

LSL_1337	-	hypothetical protein LSL_1337	Chromosome	-4.49	-4.80	-42.32	-74.10
LSL_1338	-	DNA-binding protein	Chromosome	-4.75	-5.76	-12.69	-26.08
LSL_1798	-	hypothetical protein LSL_1798	pMP118	-2.36	-3.39	-2.74	-13.06
LSL_1799	-	hypothetical protein LSL_1799	pMP118	-1.64	-3.27	-1.53	-7.96
LSL_1800	-	hypothetical protein LSL_1800	pMP118	-1.57	-3.31	-1.26	-7.72
LSL_1801	-	choloylglycine hydrolase	pMP118	-1.18	-2.32	-1.51	-7.14
LSL_1829	-	type II restriction enzyme	pMP118	-5.03	-5.18	-3.65	-3.35
LSL_1864	-	hypothetical protein LSL_1864	pMP118	-3.56	-2.16	-2.41	-2.60
LSL_1865	-	hypothetical protein LSL_1865	pMP118	-3.18	-1.88	-2.48	-2.31
LSL_1872	*pflA*	pyruvate formate-lyase activating enzyme	pMP118	-57.90	-4.29	-22.76	-4.25
LSL_1873	*pflD*	formate acetyltransferase	pMP118	-25.31	-2.18	-17.43	-2.38
LSL_1883	-	hypothetical protein LSL_1883	pMP118	-4.79	-5.94	2.05	1.95
LSL_1907	-	hypothetical protein LSL_1907	pMP118	-1.03	-3.71	-1.49	-8.07
LSL_1908	-	hypothetical protein LSL_1908	pMP118	1.16	-2.62	-1.25	-4.08
LSL_1884	-	hypothetical protein LSL_1884	pMP118	-4.54	-7.23	-404.82	-233.90
LSL_1885	-	hypothetical protein LSL_1885	pMP118	-659.12	-357.86	-1243.96	-597.30
